# Folate-conjugated near-infrared fluorescent perfluorocarbon nanoemulsions as theranostics for activated macrophage COX-2 inhibition

**DOI:** 10.1038/s41598-023-41959-9

**Published:** 2023-09-14

**Authors:** Riddhi Vichare, Caitlin Crelli, Lu Liu, Rebecca McCallin, Abree Cowan, Stefan Stratimirovic, Michele Herneisey, John A. Pollock, Jelena M. Janjic

**Affiliations:** 1 Graduate School of Pharmaceutical Sciences, School of Pharmacy, Duquesne University, Pittsburgh, PA 15282 USA; 2 Department of Biological Sciences, School of Science and Engineering, Duquesne University, Pittsburgh, PA 15282 USA

**Keywords:** Drug delivery, Imaging techniques and agents

## Abstract

Activated macrophages play a critical role in the orchestration of inflammation and inflammatory pain in several chronic diseases. We present here the first perfluorocarbon nanoemulsion (PFC NE) that is designed to preferentially target activated macrophages and can deliver up to three payloads (two fluorescent dyes and a COX-2 inhibitor). Folate receptors are overexpressed on activated macrophages. Therefore, we introduced a folate-PEG-cholesterol conjugate into the formulation. The incorporation of folate conjugate did not require changes in processing parameters and did not change the droplet size or fluorescent properties of the PFC NE. The uptake of folate-conjugated PFC NE was higher in activated macrophages than in resting macrophages. Flow cytometry showed that the uptake of folate-conjugated PFC NE occurred by both phagocytosis and receptor-mediated endocytosis. Furthermore, folate-conjugated PFC NE inhibited the release of proinflammatory cytokines (TNF-α and IL-6) more effectively than nonmodified PFC NE, while drug loading and COX-2 inhibition were comparable. The PFC NEs reported here were successfully produced on multiple scales, from 25 to 200 mL, and by using two distinct processors (microfluidizers: M110S and LM20). Therefore, folate-conjugated PFC NEs are viable anti-inflammatory theranostic nanosystems for macrophage drug delivery and imaging.

## Introduction

Macrophages are adaptive immune cells and key drivers of acute and chronic inflammation^1^ implicated in a wide range of debilitating chronic illnesses, such as diabetes^2^, cardiovascular diseases^3^, arthritis^4^, asthma^5^, and cancer^6^. These inflammatory diseases profoundly affect the quality of life and are unavoidably linked to a significant socioeconomic burden^7^. Macrophages are activated in response to various environmental cues (e.g., microbial products and activated lymphocytes) and in certain autoimmune diseases^8^. Persistent macrophage activation leads to overexpression of cyclooxygenase-2 (COX-2), a primary enzyme that catalyzes the rate-limiting step during prostaglandin E2 (PgE_2_) synthesis^9^. Activated macrophages overproduce PgE_2,_ which increases vascular permeability and is the key factor behind clinical manifestations of inflammation, such as redness, heat, swelling, and pain^10^. The production of proinflammatory cytokines, such as tumor necrosis factor-α (TNF-α), interleukin 6 (IL-6), and interleukin beta (IL-β)^11^, is also increased, which further exacerbates inflammation and pain. Inhibition of COX-2 in activated macrophages specifically decreased PgE_2_, inflammation, and pain associated with infection, injury, and inflammatory diseases.

We have reported in several animal models that macrophage modulation with a COX-2 inhibitor leads to suppression of inflammation and reduces pain-like behavior^9,12–16^. In these studies, macrophage COX-2 inhibition was achieved by a theranostic nanomedicine formulated as a nanoemulsion (NE). Theranostic nanosystems are designed to simultaneously act as therapeutic and diagnostic agents^12,17,18^. NEs are small (mean droplet diameter < 500 nm) oil-in-water emulsion droplets that are ideally suited for theranostics due to their surface-area-to-volume ratio, which allows for effective drug loading, extended release^19^, and the ability to functionalize the surface with targeting ligands and imaging moieties (metal chelates, dyes, etc.)^9,20–22^. NEs are commonly used to increase the bioavailability of poorly soluble drugs^23–27^. NEs are also easily incorporated into other dosage forms and have potential for scale-up^28,29^. Therefore, we selected NEs as our macrophage-targeted drug delivery and imaging platform.

In this and our earlier studies, we used perfluorocarbon nanoemulsions (PFC NEs) as a macrophage-directed theranostic platform. PFCs are attractive theranostic NE building blocks, as they are biologically inert and can be quantitatively detected in vivo by ^19^F MRI^30–33^. PFC NEs can thus serve as bimodal imaging agents by incorporating other types of imaging moieties, such as near-infrared fluorescent (NIRF) dyes^34–36^. We have developed multiple theranostic NEs that can carry a higher lipophilic payload (drug and/or dye) with multimodal imaging capability as well as exceptional colloidal and photostability^31,37,38^. We have also shown successful scale-up (batch size-1 L) and long-term stability of drug-loaded PFC NE^39^.

The current investigation builds on our earlier work on theranostic NEs to deliver celecoxib (CXB), a clinically used selective COX-2 inhibitor. The presented work introduces a targeting agent-folate-to NE surface for increasing the uptake in activated macrophages. Furthermore, we report a new formulation approach that resulted in higher PFC oil and drug loading over previous reports, has the ability to incorporate multiple fluorescent dyes, including a clinical grade indocyanine green (ICG) dye, and can be scaled up to 200 mL with maintained colloidal and fluorescent stability.

Overexpression of folate receptors (FR-β isoform) on activated macrophages during inflammatory diseases has been supported by compelling evidence from both preclinical and clinical studies^40,41^. Folate receptors (FRs) comprise a family of 35–40 kDa glycosylphosphatidylinositol-anchored proteins that bind with high affinity to folic acid- or folate-conjugated nanosystems (K_D_ < 1 nM)^42^. Human FR (hFR) comprises three isoforms, FR-α, FR-β, and FR-γ, with distinctive patterns of tissue distribution^43^. The feasibility of targeting the FR-β isoform to mediate macrophage-specific drug delivery is due to its unique characteristics. (1) The FR-β isoform is specifically overexpressed on activated macrophages recruited by inflammatory stimuli, not by resting macrophages. (2) The FR-β receptor is a fully functional receptor that is capable of binding the targeting ligand, mediating rapid endocytosis, and recycling back to the macrophage surface^44^. A recent study demonstrated that folate surface-conjugated liposomes selectively bind to activated macrophages in rheumatoid arthritis^45^. In another report, folate-conjugated generation-4 dendrimers preferentially accumulated in inflamed tissue by targeting the FR-β isoform expressed on activated macrophages^46^. Therefore, we selected folate as the targeting ligand for CXB-loaded PFC NEs with the goal of enhancing their uptake in activated macrophages. The present study demonstrates for the first time, to the best of our knowledge, that PFC NEs can incorporate up to three payloads (celecoxib and two fluorescent dyes) and are folate conjugated on their surface while maintaining colloidal and fluorescence stability. The present in vitro studies demonstrated that surface conjugation with folate enhanced uptake in activated macrophages and was more therapeutically effective than the nontargeted PFC NEs. The novel PFC NE formulation was also successfully manufactured on small (25 mL) and large (up to 200 mL) scales with retained colloidal properties on two different processors.

## Results

### Preparation and characterization of folate surface-decorated PFC NEs

PFCs are commonly formulated as PFC oil-in-water NEs, where PFC droplets are stabilized by surfactants^47,48^. The presented PFC NEs are based on our earlier established structure of “triphasic” PFC NE, where both PFC and hydrocarbon oil are combined in an internal phase and dispersed into surfactant-stabilized droplets in an aqueous, external phase^9,12,13,15,16,22,39,49–53^. In this study, we modified previously reported triphasic NEs by increasing the content of perfluoro-15-crown-5 ether (PCE), replacing the surfactant system, and introducing the targeting ligand folate on the PFC NE droplet surface. Folic acid is introduced to the nanoemulsion surface by adding a commercially available folate-polyethylene glycol spacer (PEG_2000_)-cholesterol conjugate to the NE oil phase during pre-emulsification (see Materials and Methods). Table 1 summarizes representative formulations of the theranostic PFC NEs that were developed to increase both imaging and drug delivery functionality as injectable nanosystems. First, we replaced the potentially sensitizing surfactant Cremophor-EL (CrEL). Despite the benefits of CrEL as a pharmaceutical vehicle to solubilize hydrophobic drugs such as CXB^54^, formulations with CrEL are reported to induce acute dose-dependent hypersensitivity reactions (e.g., complement activation-related pseudoallergy)^55,56^. Pluronic^®^ P123 (P123), which is relatively nontoxic and does not elicit allergic reactions, was adopted as a substitute for CrEL^57^. All presented formulations (Table 1) were prepared with a 4.15% w/v P105 and 0.85% w/v P123 surfactant blend, in contrast to the previously reported Kolliphor (CrEL) as the key surfactant^58^. The hydrophilic-lipophilic balance (HLB) value of the P105/P123 surfactant blend matched those of the formulations with P105/CrEL and was calculated using Eq. (1):

1 $${\text{HLB}}_{{{\text{mix}}}} = {\text{F}}_{{{\text{P123}}}} {\text{HLB}}_{{{\text{P123}}}} + {\text{ F}}_{{{\text{P1}}0{5}}} {\text{HLB}}_{{{\text{P1}}0{5}}}$$

where HLB_mix_, HLB_P123_, HLB_P105_ are the HLB values of mixed surfactants, Pluronic 123 (P123, HLB = 22) and Pluronic 105 (P105, HLB = 15), respectively, and F_P123_, F_P105_ are the weight fractions of P123 and P105.

**Table 1:** PFC NE composition table with payloads, manufacturing scale and drug loading.

Code	Scale (mL)	PCE	Drug	Folate	Dye	Droplet diameter	Poly Dispersity Index PDI	Percent drug loading
NE 1	25	+	CXB	−	DiR	128.9	0.089	76.0 ± 0.3
NE 2	25	+	−	−	DiR	128.1	0.109	–
NE 3	25	+	CXB	+	DiR	126.5	0.110	76.7 ± 2.1
NE 4	25	+	−	+	DiR	121.2	0.100	–
NE 5	175	+	CXB	−	DiR	126.5	0.110	77.2 ± 1.8
NE 6	175	+	−	−	DiR	114.1	0.076	–
NE 7	25	+	CXB	−	DiD	115.2	0.107	74.5 ± 0.9
NE 8	25	−	CXB	+	DiI/DiR	125.1	0.122	76.1 ± 0.3
NE 9	25	+	CXB	−	ICG	117.5	0.103	79.5 ± 0.1
NE 10	200	+	CXB	−	ICG	116.8	0.161	72.3 ± 0.1

**Figure 1:**
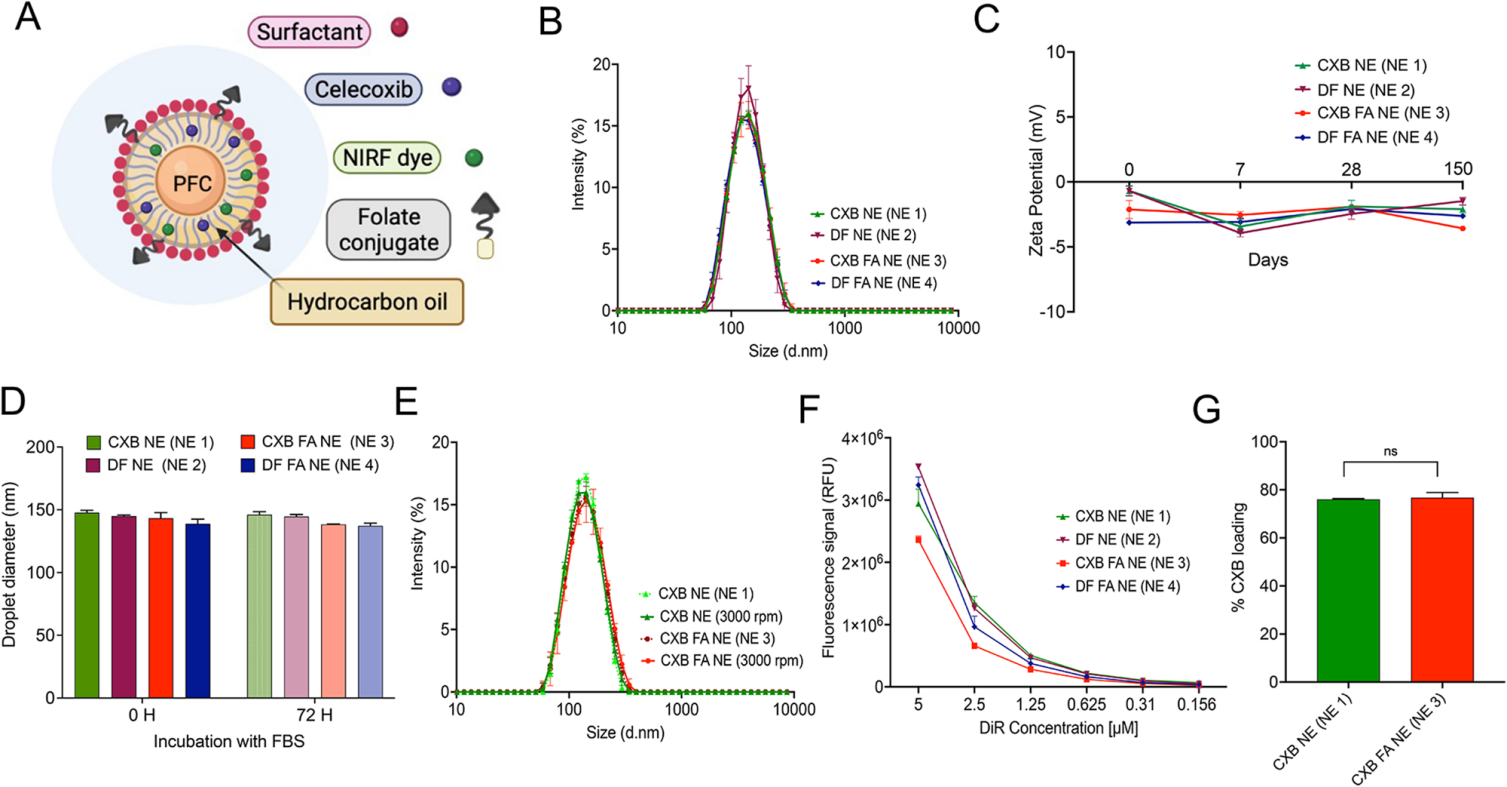
Comparative characterization of NEs with or without folate. (**A**) Schematic representation of folate-conjugated CXB NE (created with BioRender.com). (**B**) Overlay of average size distribution by intensity. (**C**) Five-month follow-up of zeta potential. (**D**) Mean droplet diameter (nm) of NEs stored for 72 h at 37 °C in 20% FBS-containing cell culture media. (**E**) Size distribution overlays of NEs before and after centrifugation at 3000 rpm for 30 min. (**F**) NIRF signal comparison performed at the same settings and compared to NEs with or without folate. (**G**) Comparison of CXB drug loading in NEs with and without folate. Data is presented as the mean ± SD (n = 3). *ns* not significant.

Second, we increased drug loading by optimizing the PFC/hydrocarbon oil content and ratio^52^. By increasing the drug content (CXB) in NEs compared to our earlier studies^12^ we aimed to reduce the overall body burden of NE for future in vivo studies. PFC NEs can serve as an imaging agent of targeted macrophages, allowing for simultaneous monitoring of the inflammatory response in vivo, which correlates to pharmacological action (e.g., pain behavior)^13,15,51^. Therefore, we also changed the PFC content and types of NIRF dyes, which would facilitate maintaining good macrophage tracking functionality with a reduced overall NE dose. With these goals in mind, the PFC oil phase (perfluoro-15-crown-5 ether, PCE) was increased from 7.8% w/v to 30% w/v. Based on previous reports, NEs with 15% w/v to 30% w/v PFC content are well suited for successful macrophage tracking in vivo by ^19^F MRI^22,59,60^. Moreover, in Phase I clinical trials, a commercially available intravenous formulation of 30% v/v PFC emulsion (VS-1000H, Celsense, USA) has demonstrated success as a ^19^F MRI diagnostic agent^47,61^. However, all these previous examples did not actively target activated macrophages. Therefore, we introduced folate to the PFC NE surface and characterized the NEs (Fig. 1A).

Table 1 shows a list of folate-decorated (FA NEs) and unmodified NEs that were produced on a small scale by manufacturing on the Microfluidizer M110S by using earlier established methods^52,53^. To incorporate the folate-PEG_2000-_cholesterol conjugate, we simply mixed it with hydrocarbon oil and transcutol during the pre-emulsification step. Importantly, the introduction of folate did not significantly change the overall droplet diameter. The size distributions of CXB NE (NE 1), DF NE (NE 2), CXB FA NE (NE 3), and DF FA NE (NE 4) tightly overlapped each other with an average droplet diameter of ~ 125 nm (Fig. 1B). The mean zeta potential values obtained for all NE formulations with or without folate remained consistent with an almost neutral zeta potential of -3 mV (Fig. 1C). These NEs are intended for parenteral administration, and their stability in the presence of biological media at body temperatures is critical for their function as drug delivery and macrophage-specific imaging systems. To model the stressors of the biological matrix, all NEs were exposed to high FBS-containing cell culture media at a body temperature of 37 °C for 72 h. No significant changes in droplet diameter were observed (Fig. 1D). To test NE stability under mechanical stress, we performed a centrifugation test, exposing NEs to 3000 rpm for 30 min for forced separation. No visual phase separation or significant changes in droplet diameter were observed after high-speed centrifugation (Fig. 1E). As NEs must serve as macrophage-specific imaging agents, fluorescence stability is essential for their in vivo use. The fluorescence signal from the embedded hydrophobic NIRF dye was consistent across all the developed NEs (Fig. 1F). Importantly, the percent drug loading was the same: 76.0 ± 0.3% for CXB NE and 76.7 ± 2.1% for CXB FA NE (Fig. 1G). The final concentration of CXB NEs with or without folate was approximately 4 mM.

### Thermal and freeze–thaw cycles for evaluating PFC NE colloidal stability

Colloidal stability and imaging properties (e.g., fluorescence) need to be continuously monitored over the lifetime of theranostic nanomedicines^37,49,62–64^.

**Figure 2:**
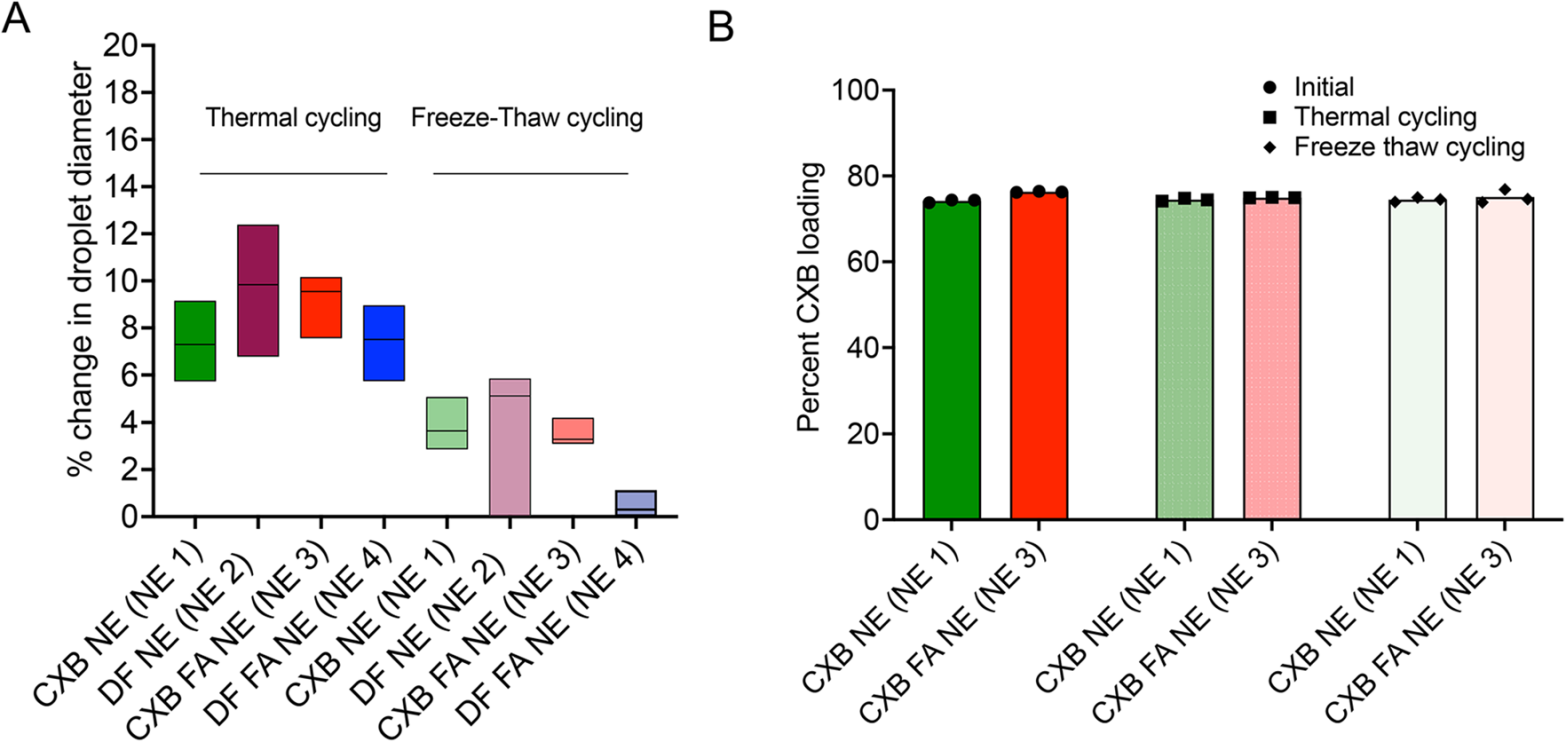
Physical stability of NEs post thermal and freeze–thaw cycling. (**A**) Calculated percent change in the droplet diameter post thermal and freeze–thaw cycles. For all the NEs, the percent change in droplet diameter was < 10%. (**B**) Comparison of percent CXB loading before and after completion of thermal and freeze thaw cycles. Data is presented as the mean $\pm$ SD (n = 3).

Table 2 presents selected quality and stability tests of PFC NEs relevant to their intended function as theranostic nanomedicines for macrophages and associated acceptance criteria for the advancement of specific formulations to in vivo studies*.* PFC NEs were subjected to cyclic fluctuations in temperature (from high temperature to low temperature) that traditionally can result in NE droplet destabilization^65^. There was less than a 10% change, as described in Table 2, in the mean droplet diameter following four thermal cycles between 50 °C (high temperature) and 4 °C (low temperature) and two freeze–thaw cycles by alternating samples between −20 and 25 °C, demonstrating the stability of NEs (Fig. 2A). Extreme temperature fluctuations may decrease drug solubility and change the percent drug loading in nanomedicines. This has direct implications for the in vivo bioavailability of CXB. The drug loading remained constant regardless of thermal and freeze–thaw cycling stress (Fig. 2B).

**Table 2:** Summary of stability tests, acceptance criteria, and evaluation methods.

Test	Specification	Evaluation method
Thermal cycling	Droplet diameter change < 10% PDI change < 0.05	Alternate between 4 and 50 °C every 24 h (4 thermal cycles)
Freeze–thaw cycle		Freeze at −20 °C for and thaw till RT 24 h (2 cycles)
Centrifugation stability		3000 rpm (30 min)
Filtration		0.22 μM pore size filter
Serum stability		72 h incubation in biological media at 37 °C
Fluorescence stability	Fluorescence emission at 700/800 nm channel yield change < 25%	NIRF Imager (Odyssey) At a set intensity and focus

### In vitro drug release from folate-conjugated PFC NE

**Figure 3:**
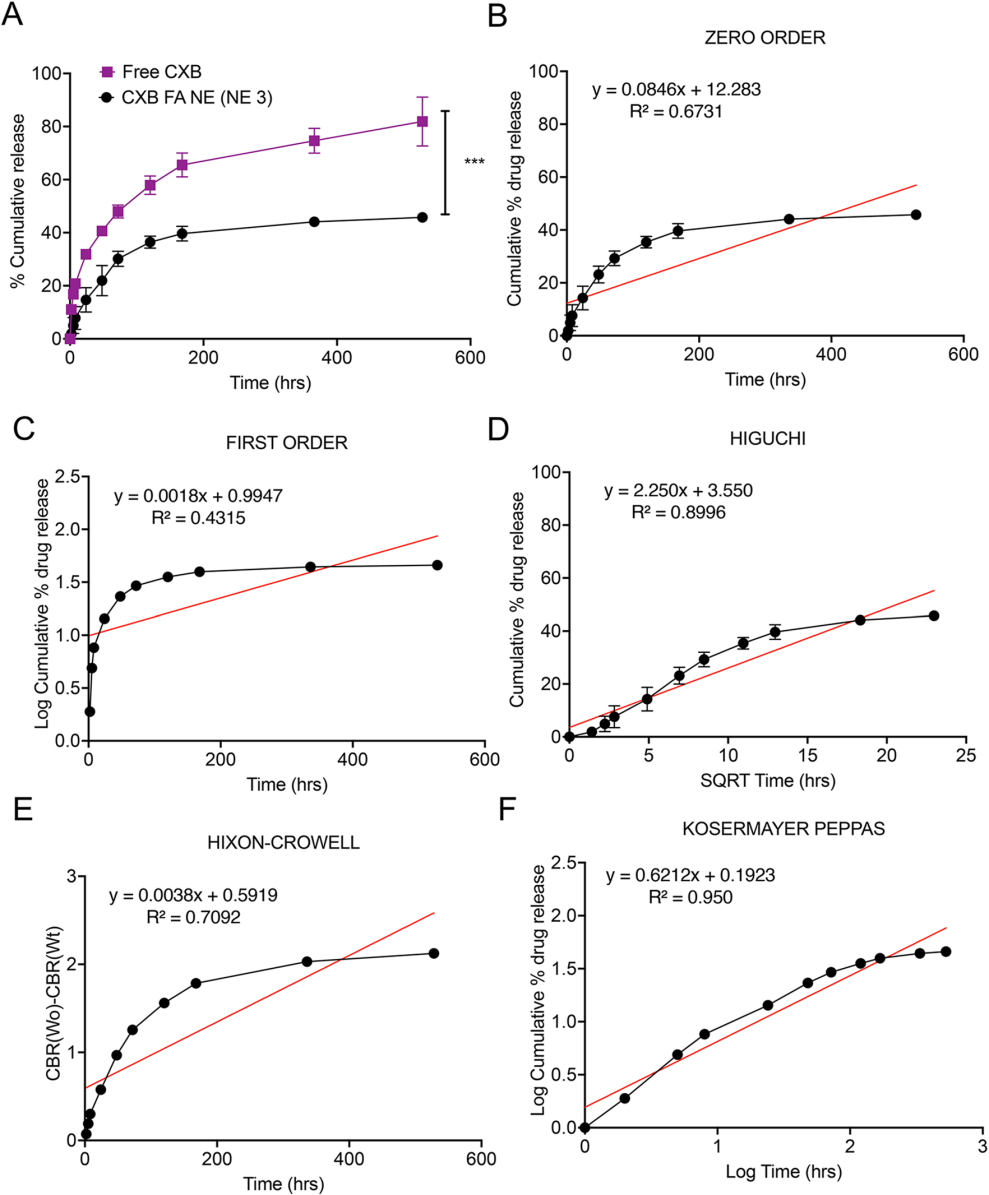
In vitro release study. (**A**) Comparison of in vitro release between CXB FA NE and CXB solution at 37 °C. The data is expressed as the mean values ± SD (n = 3). (**B**) Graphical representation of zero-order kinetic data analysis of CXB FA NE. (**C**) Graphical representation of the first-order kinetic data analysis of CXB FA NE. (**D**) Graphical representation of Higuchi kinetic data analysis of CXB FA NE. (**E**) Graphical representation of Hixon–Crowell kinetic data analysis of CXB FA NE. (**F**) Graphical representation of the Kosermayer–Peppas kinetic data analysis of CXB FA NE.

The in vitro release behavior of CXB from folate-conjugated PFC NE was studied using a dialysis membrane at 37  ± 0.5 °C. The percentage cumulative release of CXB from CXB FA NE was only 1.89 ± 1.5%; however, CXB solution relatively demonstrated a burst release pattern, as 11.06 ± 0.203% of CXB was released within 2 h at 37  ± 0.5 °C (Fig. 3A). The release pattern from CXB FA NE was more sustained, as only 39.64 ± 2.7% of CXB was released at the end of the week. A cumulative release of 65.5 ± 4.5% was achieved from the CXB solution during the same duration. At the end of the study, the percent cumulative release of CXB from NE and the drug solution was 45.7 ± 1.64% and 87.2 ± 0.27%, respectively. The release data were fitted into mathematical models for quantitative interpretation of release mechanisms and kinetics^66^.

The kinetics of dissolution were fitted to the zero-order, first-order, Higuchi, Hixson–Crowell, and Korsmeyer-Peppas models (Fig. 3B–F). The best model was determined based on the value of the correlation coefficient (R^2^). The CXB FA NE followed the Korsmeyer-Peppas model (R^2^ = 0.950). The mechanism of drug release was studied using the Korsmeyer-Peppas Eq. (2):

2 $$\frac{Mt}{M\infty }=k \times {t}^{n}$$

where Mt is the cumulative amount of drug released at time t, M∞ is the amount of drug release at infinite time, k is a constant characteristic of the drug–polymer system, and n is the exponent of release indicative of the nature of the drug release mechanism. Furthermore, the value of n was determined to be 0.361, characteristic of Fickian diffusion (0 < n < 0.45).

### Nanoemulsion effect on macrophage viability

**Figure 4:**
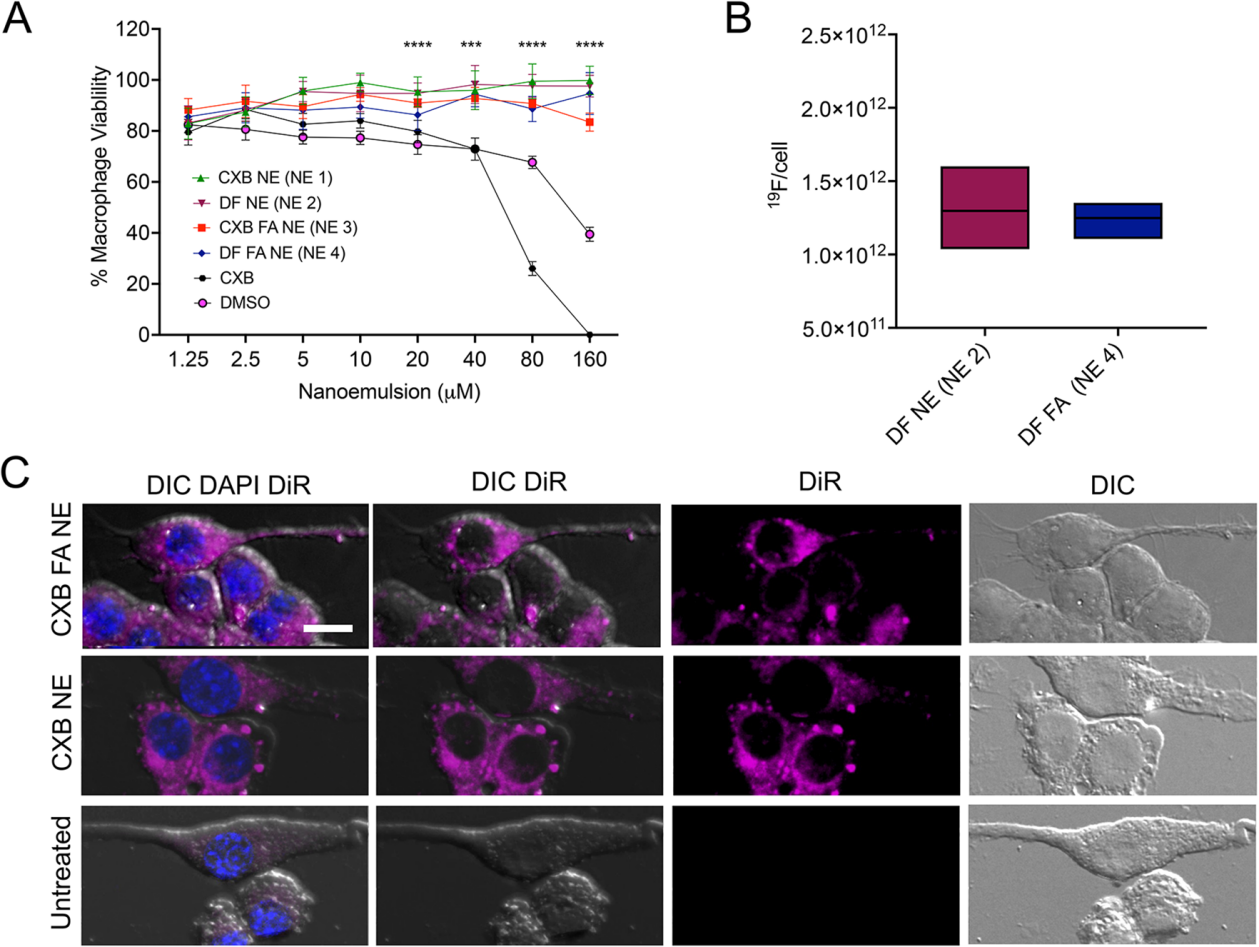
Cell viability, ^19^F NMR and fluorescence microscopy. (**A**) Macrophages were exposed to NE 1, NE 2, NE 3, NE 4, CXB in DMSO, and free drug vehicle: DMSO for 24 h. Assay performed via ATP based CellTiter-Glo 2.0 (n = 6). (**B**) ^19^F/cell was quantified in NEs with or without folate (n = 3). (**C**) Fluorescence microscopy of Raw 264.7 macrophages exposed to CXB NE (NE 1) and CXB FA NE (NE 3) for 6 h. Cells were stained for nuclei (DAPI, blue) and DiR labeled NEs (Purple). Panels viewed left to right show the overlay of DIC DiR (NIRF) and DAPI, DIC DiR, DiR alone and DIC alone. Scale bar: 10 μm. ****p < 0.00005.

The developed NEs were tested using the CellTiter-Glo luminescent assay to determine RAW 264.7 macrophage viability after 24 h of exposure. The developed NEs did not exert cytotoxic effect on macrophages and thus were safe for performing further in vitro studies. However, the CXB free drug in DMSO and volume-matched DMSO exhibited dose-dependent toxicity (Fig. 4A). The viability of macrophages treated with CXB solution from 10 to 160 μM dropped significantly compared to CXB NE or CXB FA NE. For definitive proof of cell viability, a CellTox™ Green assay was performed, which measures changes in cell membrane integrity as an indication of cell death. This assay intends to detect loss of cellular and nuclear membrane integrity as a function of the fluorescent signal. Macrophages treated with varying concentrations of CXB FA NE and volume-matched DF FA NE showed no compromise in membrane integrity compared to CXB free drug solution (Supplementary Fig. 1A). Cytotoxicity study was repeated on lipopolysaccharide (LPS)-activated macrophages (Supplementary Fig. 1B).

### Activated macrophage uptake studies

The ability of the presented NEs to demonstrate cell labeling was confirmed with ^19^F NMR spectroscopy using lysed cell pellet from RAW 264.7 macrophages. For both the NEs with or without folate, suitable levels of NE uptake for in vivo MRI (i.e., > 10^11 19^F/cell) were achieved in 6 h of incubation (Fig. 4B). The PCE-labeled cells showed a major peak at − 92.45 ppm and the − 76.00 ppm peak was from TFA reference added to lysed cell pellet (Supplementary Fig. 2A, B). The integrated areas under these two peaks were used to calculate the mean ^19^F/cell, often ranging from 10^11^ to 10^13 19^F/cell^67^. The cellular uptake of DiR-labeled NE droplets was qualitatively observed using epi-fluorescence microscopy (Fig. 4C). Both the DiR-labeled NEs with or without folate were taken up by macrophages. However, as the control group was not exposed to NE, no NIRF signal was observed. The fluorescence from the DiR showed a punctate pattern in macrophages. Confocal microscopy with z-series optical sectioning reveals that the punctate DiR fluorescence resides within the cytoplasm of the cell (Supplementary Fig. 3).

**Figure 5:**
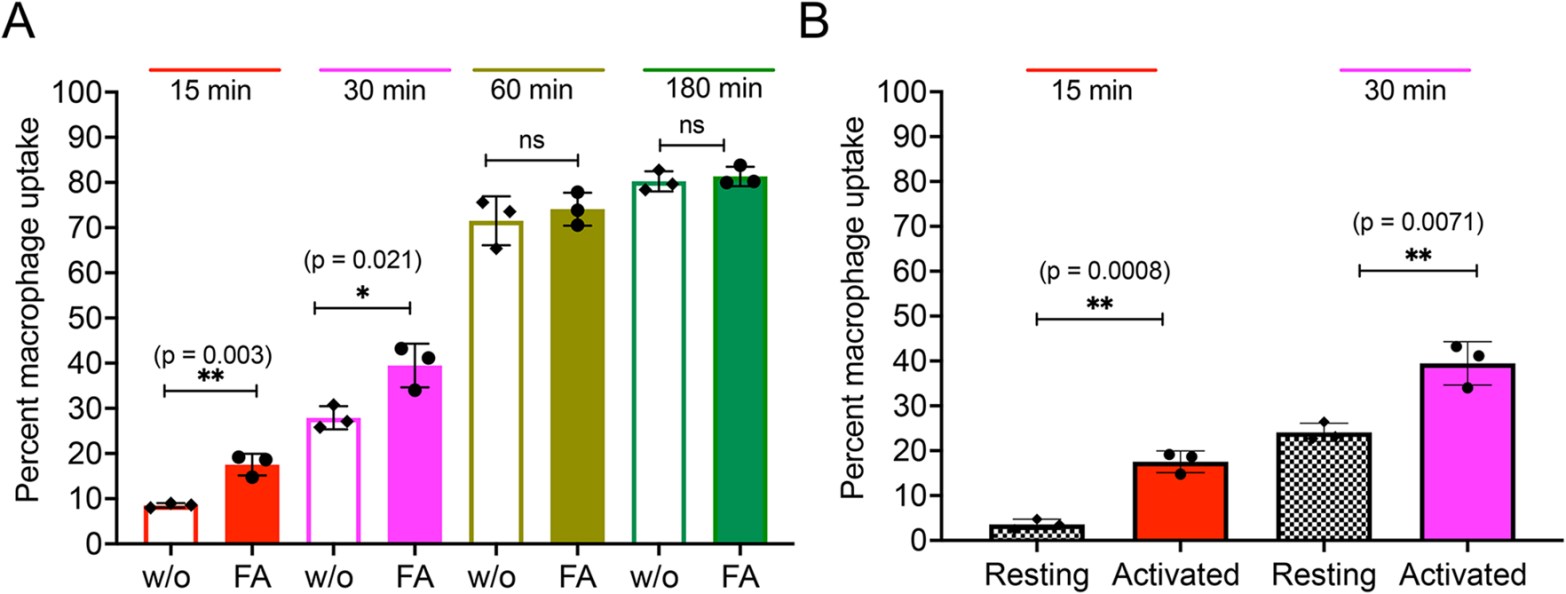
Flow cytometry analysis of lipopolysaccharide (LPS)-activated RAW 264.7 macrophages. (**A**) Quantitative analysis of the fluorescence intensity of DiR-labeled CXB NE (NE 1) and CXB FA NE (NE 3) in activated macrophages. (**B**) Comparison of cellular uptake of CXB FA NE (NE 3) in activated and resting macrophages. The data is shown as the mean ± SD (n = 3/group), and 40,000 cells were counted. For gating information and dot plots refer Supplementary Fig. 4. *ns* not significant, *p < 0.05, **p < 0.005.

Furthermore, quantitative uptake studies were conducted on LPS-activated macrophages by performing flow cytometry. We compared the uptake of CXB FA NE to that of nontargeted CXB NE. As shown in Fig. 5A, time-dependent macrophage uptake of NEs was observed. As hypothesized, the cellular uptake of CXB FA NE was higher (17.5 $\pm$ 2.5%) than that of CXB NE (8.5 $\pm$ 0.4%) after 15 min of exposure. The uptake of CXB FA NE was statistically significant compared with CXB NE even after 30 min of exposure. However, after 1 h of exposure to the NEs, macrophage uptake almost reached saturation, and no significant differences were observed at later timepoints. Furthermore, we evaluated the uptake differences between LPS-activated macrophages and resting macrophages, choosing early time points (15 min and 30 min), since folate-decorated NEs are designed to be primarily taken up by activated macrophages but not by resting macrophages. The uptake of CXB FA NE in activated macrophages was approximately five times and two times higher than that in resting macrophages at 15 min and 30 min, respectively (Fig. 5B).

**Figure 6:**
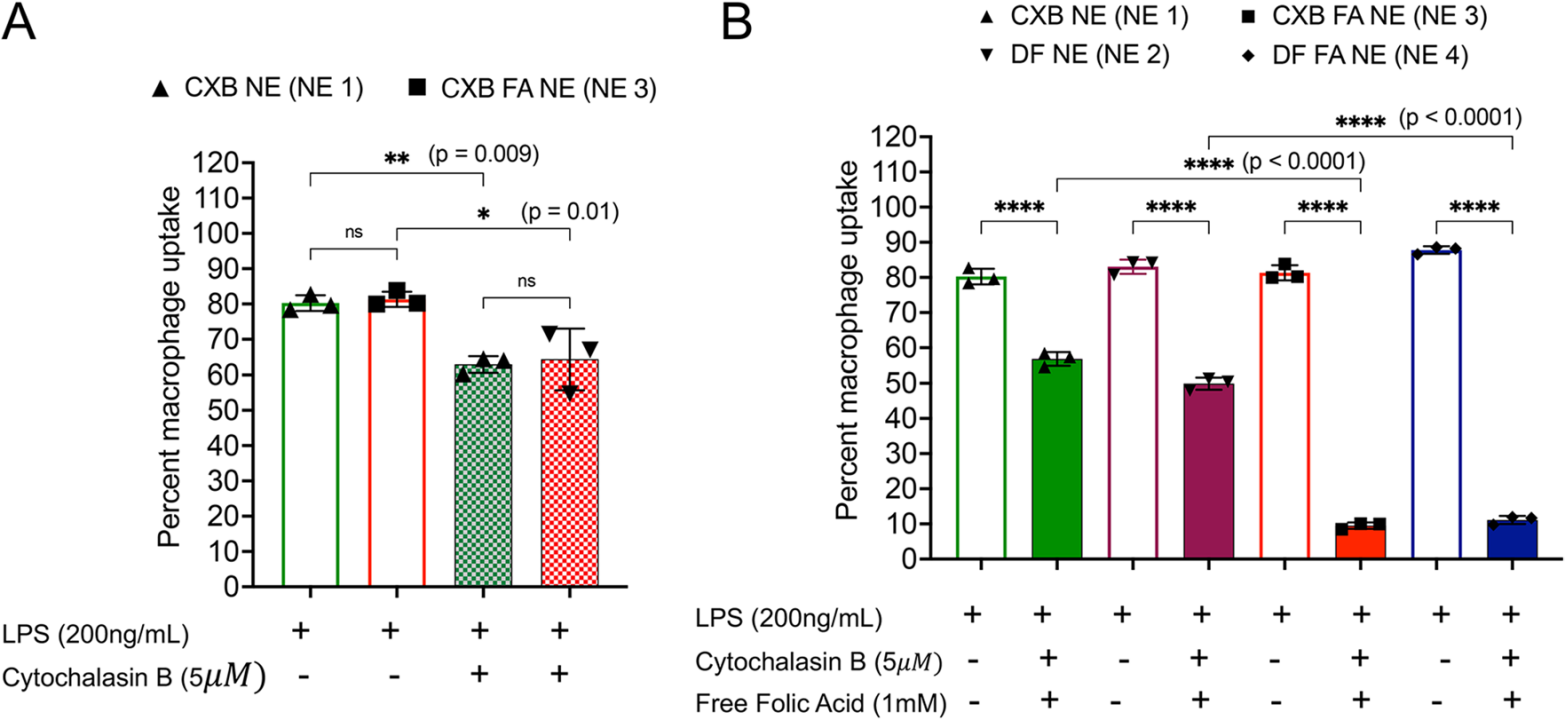
Activated macrophage uptake studies for drug loaded and drug free NEs with or without folate. (**A**) Comparison of uptake in the absence and presence of the phagocytosis inhibitor cytochalasin B (cyto B, 5 $\upmu$M). (**B**) Comparison of uptake by activated macrophages in the presence of 1 mM FFA and 5 $\upmu$M ctyo B. The data is shown as the mean ± SD (n = 3/treatment condition), and 40,000 events were counted. *p < 0.05, **p < 0.005, ****p < 0.00005.

To elucidate the uptake mechanisms of activated macrophages, the phagocytosis inhibitor cytochalasin B (cyto B, 5 μg/ml) was used alone or concomitantly with excess mol of free folic acid (FFA, 1 mM). In the presence of cyto B alone, CXB NE and CXB FA NE showed an ~ 20% decrease in uptake compared to the controls (macrophages treated without cytochalasin B) (Fig. 6A). This shows that internalization of PFC NEs by activated macrophages occurs through an energy-dependent active process such as phagocytosis^53^. A competitive binding assay was performed to test uptake by receptor-mediated endocytosis. Activated macrophages were treated with higher concentrations of FFA concomitantly with cyto B. The presence of FFA in the medium acts as a competitor to saturate the expressed FR- receptors on activated macrophages^68^. The results demonstrated a significant decrease in CXB FA NE and DF FA NE in the presence of excess free folic acid as opposed to CXB NE and DF NE (Fig. 6B), suggesting that FA-conjugated NEs bind to FR on activated macrophages in a receptor-specific manner. Note that the chosen concentration of FFA is significantly higher than the normal concentration seen in human physiological plasma (50 nM)^69^.

### Effect of folate-conjugated PFC NE on the release of inflammatory mediators

**Figure 7:**
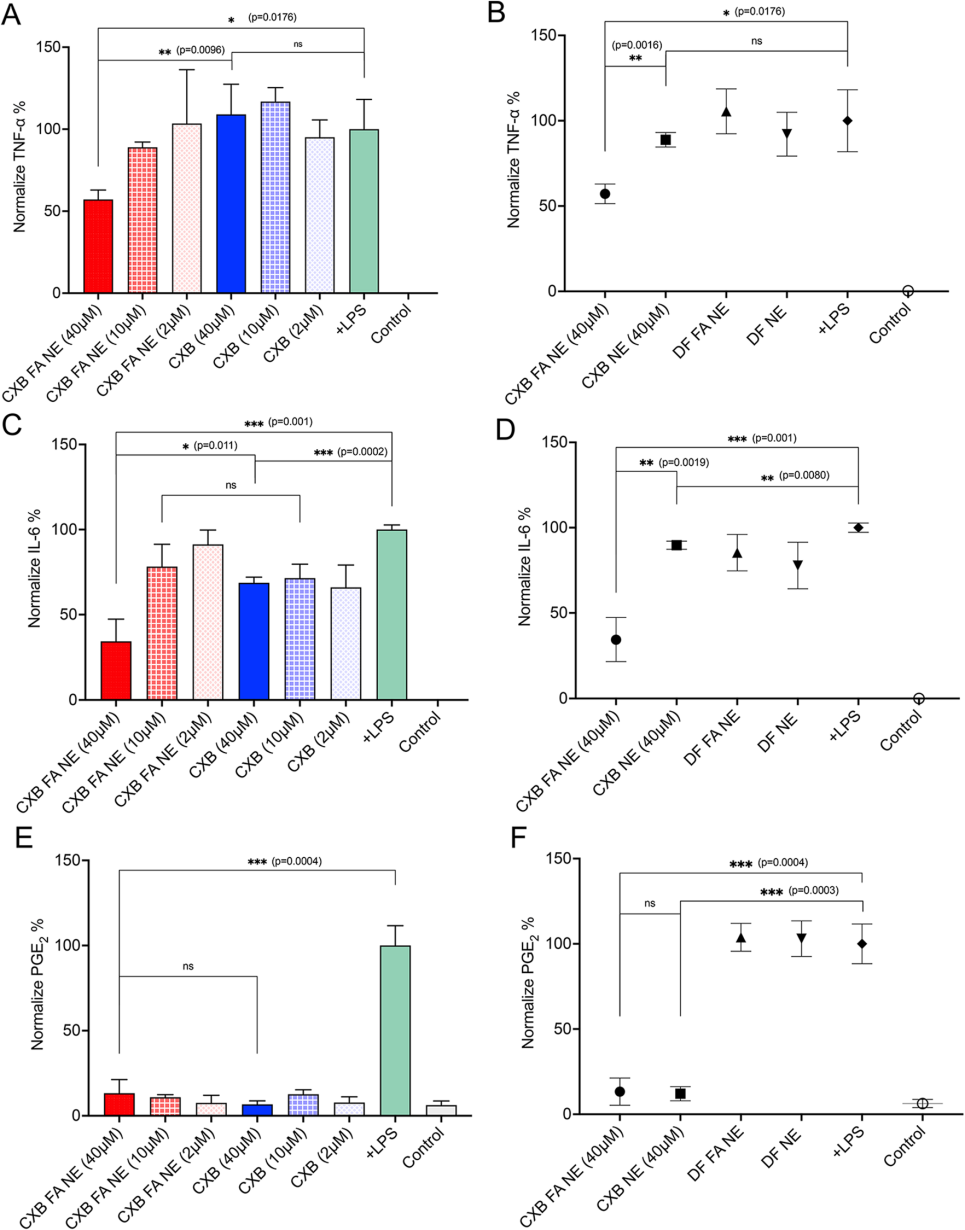
The pharmacological response of NEs with or without folate and CXB solution on activated macrophages. (**A**) Inhibition of TNF-α release from LPS-activated macrophages exposed to CXB FA NE and CXB-free solution at different concentrations. (**B**) Comparison of TNF-α inhibition in activated macrophages exposed to CXB FA NE and CXB NE at a single (40 µM) concentration. (**C**) Inhibition of IL-6 release from LPS-activated macrophages exposed to CXB FA NE and CXB-free solution at different concentrations. (**D**) Comparison of IL-6 inhibition in activated macrophages exposed to CXB FA NE and CXB NE at a single (40 µM) concentration. (**E**) PgE2 inhibition from activated macrophages exposed to CXB FA NE and CXB-free solution at different concentrations. (**F**) Comparison of PgE2 inhibition from LPS-activated macrophages exposed to CXB FA NE and CXB NE at a single (40 µM) concentration. Each bar represents the mean $\pm$ SD (n = 3, *independent cultures*). *ns* not significant, **p < 0.005, ***p < 0.0005.

The anti-inflammatory effects of CXB NE, DF NE, CXB FA NE, and DF FA NE were evaluated on LPS-activated RAW 264.7 macrophages by ELISA. The efficacy of folate-conjugated NE (CXB FA NE) to suppress the release of proinflammatory cytokines was compared to a free CXB solution, which was analyzed at three different concentrations (40, 10, 2 μM). The level of cytokine release from activated macrophages was significantly higher than that in the untreated/control group. A concentration-dependent decrease in TNF-α release was observed with CXB FA NE. TNF-α relative to activated macrophages decreased by 43% at a concentration of 40 μM but only by 12% at a concentration of 10 μM. However, this dose dependency was absent in the CXB solution-treated groups. Interestingly, CXB solution, even at a higher concentration (40 μM), failed to suppress the release of TNF-α from activated macrophages (Fig. 7A). To evaluate the therapeutic benefit of folate conjugation, CXB FA NE was compared against CXB NE. In contrast to the CXB FA NE (40 μM) group, the CXB NE treatment group at equivalent concentrations showed no decrease in TNF-α (Fig. 7B). Similarly, a concentration-dependent decrease in IL-6 was observed with CXB FA NE, as concentrations of 40 μM and 10 μM resulted in 66% and 29% decreases, respectively. However, the CXB solution resulted in a 31% decrease in IL-6 levels at 40 μM (Fig. 7C). For CXB NE (40 μM), only an 11% decrease in IL-6 cytokine release was observed, which was significantly lower than that of CXB FA NE (Fig. 7D). In activated macrophages, PgE_2_ serves as a marker for the upregulated biological activity of COX-2. No differences were observed when CXB FA NE was compared to CXB solution and CXB NE (Fig. 7E, F) for levels of PgE_2_ suppression. However, a more than 80% reduction in PgE_2_ levels was observed with folate-conjugated NEs. DF NE and DF FA NE did not exert any anti-inflammatory effects on the activated macrophages.

### Large-scale manufacturing

**Figure 8:**
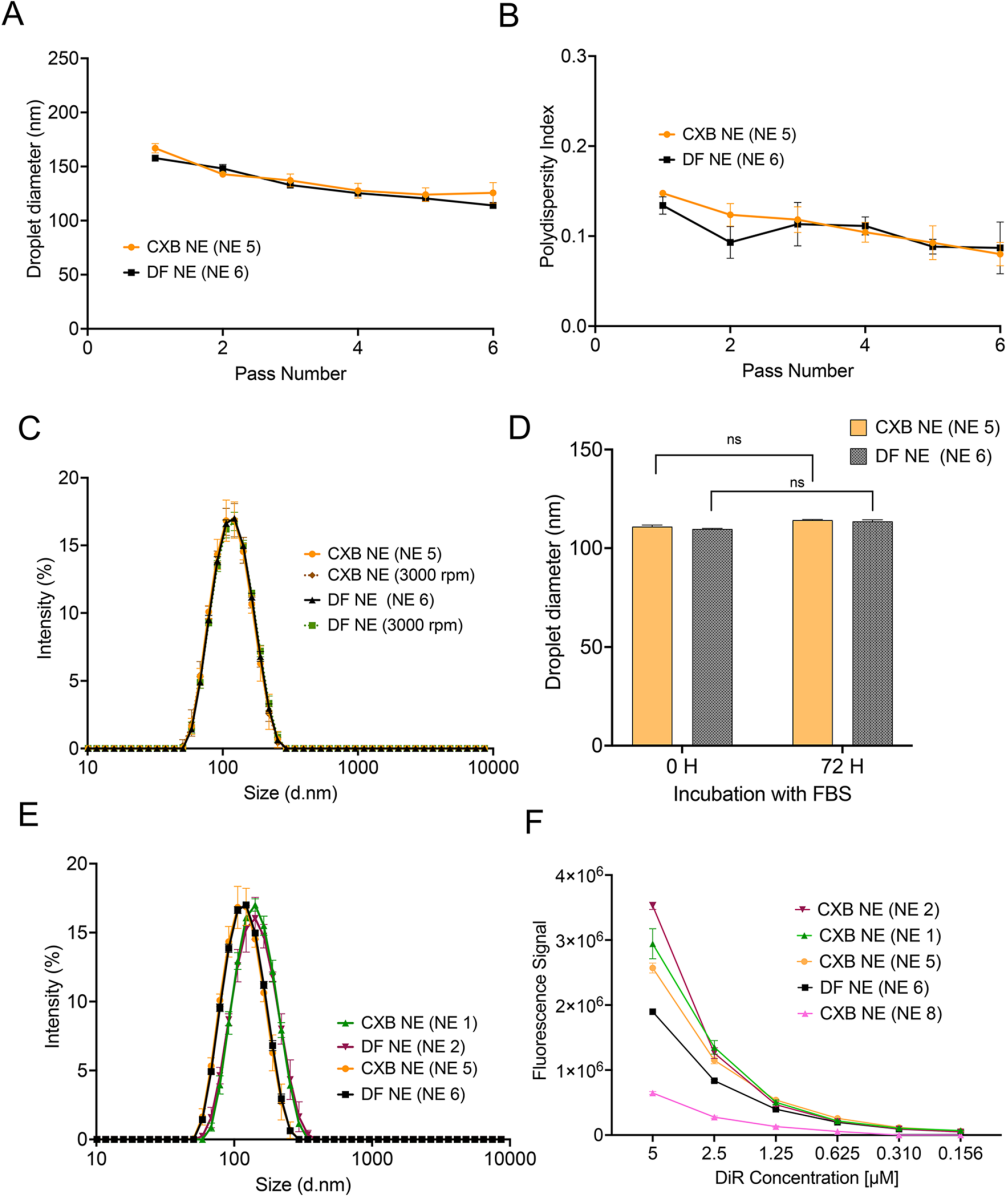
Characterization of large-scale NEs with or without CXB (175 mL) and comparison with small-scale NEs (25 mL). (**A**) Effect of the number of passes on the droplet diameter of large-scale NEs. (**B**) Effect of the number of passes on the polydispersity index (PDI) of large-scale NEs. (**C**) Overlay of size distribution before and after centrifugation at 3000 rpm for 30 min. (**D**) Droplet diameter of NEs were monitored for 72 h at 37 °C and diluted in 20% FBS-containing cell culture media. (**E**) Size distribution by intensity of NEs with or without CXB produced on a large scale compared to that produced on a small scale. (**F**) NIRF signal comparison was performed at the same settings and compared to a small-scale NE 8 with reduced PFC concentration. Data is presented as the mean $\pm$ SD (n = 3).

We also demonstrated the feasibility of manufacturing triphasic NEs on a large scale (175 mL), 7 times greater than the small lab-scale batch (25 mL) using the LM20 microfluidizer. All the manufactured large-scale NEs with different dyes are summarized in Table 1. With increased passes, a gradual decrease in mean droplet diameter (Fig. 8A) and PDI (Fig. 8B) was observed for CXB NE (NE5) and DF NE (NE6). The large-scale NEs also showed excellent colloidal stability under stress from centrifugation (Fig. 8C) and when exposed to biological medium (Fig. 8D). When comparing the large-scale batch with the small-scale batch, an overlapping size distribution was observed irrespective of the different processors (Fig. 8E). All the formulated NEs displayed a narrow size distribution with a PDI of less than 0.15 (Table 1). The fluorescent signal from NIRF dyes, such as DiR (748 nm/780 nm), was compared among the developed NEs to assess consistent manufacturing. The presence of CXB, high PCE content, P105/P123 micelle solution, and a large processing volume did not alter the fluorescence intensity for the developed NE pairs (Fig. 8F). Interestingly, with an increase in the PCE content in the new formulation, an enhanced NIRF fluorescent signal was observed even though the dye concentration remained consistent. HPLC analysis showed no significant difference in CXB loading between small- and large-scale NEs, addressing a critical challenge faced during successful scale-up (Supplementary Fig. 5A).

## Discussion

The infiltration of activated macrophages is positively correlated with the severity of several chronic inflammatory diseases^70,71^. Indeed, depletion of activated macrophages leads to the resolution of chronic inflammation and pain. Nonspecific macrophage depletion techniques such as leukocytapheresis can mitigate the symptoms of chronic inflammation but are associated with side effects^72^. Therefore, research has focused on developing therapeutics that solely target activated macrophages without compromising the function of resting macrophages. The ability of FR-β-targeted imaging agents to selectively deposit in inflamed tissues is particularly tested in human clinical trials^73,74^. Based on this concept, our group has developed folate-conjugated theranostic NEs for delivering CXB to counter inflammation and pain. The developed NEs also combine a ^19^F MRI probe (PFC) and NIRF dye to provide high diagnostic specificity for inflammatory diseases.

The surface conjugation of PFC NE with a folate ligand did not alter the droplet diameter, PDI, or zeta potential. A comparable zeta potential between NEs with or without folate is probably due to the weak negatively charged properties of FA-PEG_2000_-cholesterol^75^. PFCs are dense transparent oils with specific weights > 1.7 g/mL and can affect the Ostwald ripening rate. Ostwald ripening is a process based on the mass-transfer phenomenon, in which the gradual growth of large NE droplets is observed at the expense of smaller droplets. This eventually leads to NE droplet destabilization. Therefore, NE droplet diameter was recorded as an indicator of colloidal stability in the presence of stressors. Temperature fluctuations not only increase the rate of Ostwald ripening but also alter viscosity and interfacial tension^76^. On the other hand, the instability in NEs during freeze–thaw cycles can be due to different physicochemical phenomena, such as oil crystallization, changes in electrostatic interactions, or altered conformation of the interfacial layer. The process also induces the formation of small ice crystals, which subsequently grow, causing an increase in NE droplet size^77,78^. Under physical stressors, the increase in droplet diameter for PFC NEs with or without folate was well within specifications (Table 2). Additionally, the presence of proteins, salts, or nutrients did not alter the colloidal stability, making them suitable for in vivo studies. This is in parallel with our previously published data, where the NEs stabilized with pluronic-based surfactants decreased protein adsorption on PFC-NEs^52^.

The sustained in vitro release of CXB from CXB FA NE could be attributed to the encapsulation of the drug in the oil core of NE. When compared to the CXB solution, CXB FA NE showed a slow but consistent release of CXB. Based on mathematical modeling, CXB release from NE was primarily governed by diffusion. To diffuse into the aqueous medium, the drug molecules must overcome two barriers. First, the drug molecule should diffuse across the hydrophobic (oil) core and second, through the droplet surfactant interfacial layer^79^. The sustained release of CXB in targeted macrophages results in prolonged inhibition of macrophage-associated COX-2 and improves pain behavior^9^. Future in vivo studies are required to test this effect for the formulations presented here.

Nanomedicine toxicity primarily depends on the (i) composition of nanomaterials, (ii) size of nanoparticles, and (iii) the type of cell exposed^80^. For in vitro studies, we used highly proliferative and FR-expressing RAW 264.7 macrophages. The presented NEs were nontoxic, as cells remained metabolically active without any loss of cell membrane integrity even when exposed to the highest concentration of drug-loaded NEs. Although the macrophage uptake of CXB FA NE and CXB NE almost reached saturation within 60 min of exposure, significantly higher uptake of CXB FA NE was recorded at the initial time points. In a way, time-dependent saturation of activated macrophages suggests that prolonged exposure may not be necessary, and that folate-conjugated PFC NEs may be rapidly cleared from the bloodstream. Indeed, under physiological conditions, an intravenously administered folate-conjugated tracer ([^18^F]fluoro-PEG-folate) showed stable uptake in inflamed joints within approximately 60 min post injection^81^. The uptake of folate-conjugated NE was significantly higher in activated macrophages than in resting macrophages. This indicates the functionality of the folate ligand to specifically bind to the FRs. Consequently, the uptake of CXB FA NE and DF FA NE in the presence of excess FFA and cyto B showed an eightfold decrease in uptake. However, under the same conditions, only a twofold decrease in uptake was observed for CXB NE and DF NE. We interpret this decrease in uptake of folate-conjugated NEs by activated macrophages to be due to saturation of FRs in the presence of excess FFA.

Our results show that folate-conjugated PFC NEs can enable higher uptake in activated macrophages by phagocytosis as well as receptor-mediated endocytosis, paving the way for future in vivo studies. Increased concentrations of TNF-α and IL-6 are directly correlated with the extent of inflammatory insult in several diseases^11^; thus, their decreased levels invariably aid in the resolution of inflammation and pain. The observed higher uptake correlated with an increased suppression of LPS-induced proinflammatory cytokines such as TNF-α and IL-6 with CXB FA NE as opposed to CXB NE.

A major hurdle towards the clinical translation of targeted nanomedicines is the complexity of their composition, leading to challenges in manufacturing^82^. The presented PFC NEs were scalable, and the process was transferable from one processor to another. Despite utilizing different equipment (M110S for small scale and LM20 for large scale), all batches were comparable and reproducible. Although we reported a positive correlation between an increase in the percent diameter and the PFC content^52^, the droplet diameter was less than 150 nm for newly formulated NEs with higher PCE content.

Ohguchi et al.^83^ reported that NEs conjugated with 0.03 mol% folate ligand resulted in fivefold greater uptake in FR ( +) KB cells, while only a 3.3-fold increase was observed with 0.24 mol%. In another report, folate-conjugated micelles with 75% folate ligand density resulted in higher uptake by RAW 264.7 macrophages than micelles with a lower percentage of folate ligand^68^. The presented study employs 0.1 mol% of folate ligand and does not optimize the folate ligand density on the NE surface, which is one of the limitations of this work. However, 0.1 mol% of folate ligand did show favorable outcomes, setting the stage for future studies. In summary, to the best of our knowledge, this is the first study that amalgamates folate-surface conjugation, bimodal diagnostic agents, and the incorporation of an NSAID, celecoxib, in a single functional PFC NE.

## Materials and methods

### Materials

Celecoxib (CXB) was purchased from Sigma-Aldrich (St Louis MO, USA). Miglyol 812 N was purchased from CREMER OLEO Product Division, Hamburg, Germany. 2-(2-Ethoxyethoxy)-ethanol (Transcutol, E1022) was obtained from Spectrum, NJ, USA. Pluronic P105 and Pluronic P123 were obtained from United States Biological, Salem, MA, USA and Sigma Aldrich, St. Louis, MO, USA, respectively. Cholesterol-PEG_2000_-folate was obtained from NanoSoft, NC, USA. Perfluoro-15-crown-5 ether was purchased from ExFluor Inc., Texas. Near infrared fluorescent (NIRF) dyes such as DiR (748 nm/780 nm) were purchased from Invitrogen, DiI (549 nm/565 nm) was purchased from Life Technologies, and ICG (789 nm/814 nm) was purchased from Sigma-Aldrich (St Louis MO, USA). The CellTiter-Glo Luminescent Cell Viability Assay kit and CellTox Green cytotoxicity assay kits were purchased from Promega Corporation, Madison WI, USA. Enzyme-linked immunosorbent assay (ELISA) kits for prostaglandin E2 were purchased from Cayman Chemical Company, Ann Arbor, MI, USA; mouse TNF-α was purchased from R&D Biosystems (catalog no. DY410-05), Minneapolis, MN, USA; and mouse IL-6 (catalog no. 88-70-64-88) was purchased from Invitrogen, Waltham, MA, USA. An adherent mouse macrophage cell line (RAW 264.7) was obtained from the American Type Culture Collection (ATCC TIB-71), Rockville, MD, USA, and cultured according to the instructions. For cell culture experiments, Dulbecco's Modified Eagle Medium (DMEM; Gibco-BRL, Rockville, MD, USA) and RPMI-1640 folate-free media (Thermo Fisher Scientific, Waltham, MA, USA) were supplemented with 10% fetal bovine serum (FBS ATCC 3020-20). Cytochalasin B and folic acid were purchased from Sigma Aldrich, St Louis, MO, USA.

## Methods

### Nanoemulsion development: small-scale nanoemulsion manufacturing (25 mL)

Nanoemulsions were manufactured following previously reported procedures^58^, with certain modifications. Briefly, the M110S microfluidizer (Microfluidics Corporation, Westwood, MA) chamber was iced for 1–2 h prior to manufacturing. A pre-emulsion was formed prior to processing in the microfluidizer. Briefly, CXB (50 mg) was dissolved in miglyol 812 N and a co-solubilizer transcutol by continuously stirring overnight. For folate-conjugated nanoemulsions, 0.1% w/v commercially available cholesterol-PEG_2000_-folate was first dissolved in transcutol before adding to the miglyol 812 N. Next day, near infrared fluorescent (NIRF) dyes were incorporated into miglyol 812 N alone (for a DF NE) or predissolved CXB in miglyol 812N and transcutol solution (for a CXB NE). To incorporate ICG into the oil phase, it was first tethered to sterylamine via an ion pair interaction as reported earlier^84^. Briefly, ICG and sterylamine are premixed in DMSO before addition to the oil phase. Then, PCE was added to the mixture and vortexed for a minute. The final concentration of the micelle solution in PBS was 5% w/v, where 4.15% w/v was P105 and 0.85% w/v was P123. The pre-emulsion was vortexed at low speed to avoid foaming and then poured into the M110S inlet reservoir. The pre-emulsion was processed for 30 pulses (6 passes) at an inlet air pressure of ∼80 psi and an operating liquid pressure ∼17,500 psi before the final nanoemulsion product was released from the outshoot. The small-scale CXB NE (NE 1), DF NE (NE 2), CXB FA NE (NE 3), and DF FA NE (NE 4) were reproduced on M110S microfluidizer three times by different operators (Supplementary Fig. 6A–D).

### Large-scale nanoemulsion manufacturing (up to 200 mL).

The LM20 microfluidizer (Microfluidics Corporation, Westwood, MA) chamber was iced for 1–2 h prior to processing the pre-emulsion into an NE. Dye was added into miglyol 812 N to create DF NE. To create a CXB NE, dye was added to predissolved celecoxib in miglyol 812 N solution with transcutol. The dyes were mixed with a stir bar for approximately 15 min. Perfluorocarbon and micelles were added and mixed with an immersion blender (Cuisinart, CT, USA) for five 1-s pulses. The microfluidizer was primed with 25 mL of micelle to flush the system. The pre-emulsion was then immediately poured into the LM20 inlet. The pre-emulsion was passed through the system five times at pressure settings ranging from 18,000 to 20,000 psi. Droplet diameter was monitored at each pass on a Zetasizer Nano (Malvern Instruments, Worcestershire, UK).

### Colloidal characterization of the nanoemulsion

Nanoemulsions were characterized utilizing dynamic light scattering (DLS) to obtain hydrodynamic diameter (nm), polydispersity index (PDI), and zeta potential (mV) measurements on Zetasizer Nano (Malvern Instruments, Worcestershire, UK). DLS operating parameters were as follows: refractive indices of material and dispersant were 1.59 and 1.33 respectively; viscosity of the dispersant, 0.8872 cP; temperature, 25 °C; run time for each run 10 s; and 173° back scatter angle. All the formulated nanoemulsions were diluted 1:40 v/v in deionized water prior to any characterization by DLS. All the diluted NEs samples had count rate of between 250 and 280 kcps.

### Centrifugation and filtration studies

Nanoemulsions were centrifuged at 3000 rpm for 30 min at ambient temperature (Labnet Prism R Refrigerated Micro-Centrifuge). For the filtration test, nanoemulsions were filtered through a 0.22 µm pore size mixed cellulose ester syringe filter (Merck Millipore Ltd. USA). Physicochemical characteristics (hydrodynamic diameter and polydispersity) were evaluated after the completion of the tests. All the sample were diluted 1:40 v/v in DI water before particle size measurements.

### Thermal cycling

The developed NEs were subjected to heat/cooling cycles to assess their stability at extreme temperatures. The heating/cooling cycle was performed according to Gumeiro et al.^85^ with certain modifications. Four cycles (8 days) were performed between an oven temperature of 50 °C and refrigerator temperature of 4 °C. Physicochemical characteristics (droplet size, polydispersity, and drug loading) were evaluated before and after the completion of cycles.

### Freeze–thaw cycling

The developed NEs were stored at a temperature between −20 and 25 °C for two cycles (4 days)^86^. Physicochemical characteristics (droplet size, polydispersity, and drug loading) were evaluated before and after completion of cycles.

### Serum stability

The size distribution of nanoemulsions was measured over time after incubation in water and biological media at 37 °C for a time span of 72 h. Nanoemulsions were diluted 1:40 v/v in 20% fetal bovine serum (FBS) in Dulbecco’s Modified Eagle’s Medium (DMEM) and stored at 37 °C for 72 h. DLS measurements were obtained at the beginning and end of 72 h of incubation.

### Near infrared fluorescent (NIRF) imaging

NIRF imaging of nanoemulsions was performed on the Li-COR Odyssey. Serial dilutions of nanoemulsion and deionized water were prepared in a range of 1:5 to 1:160 v/v. Dilutions were transferred to a clear 96-well plate in triplicate, which was measured on the Li-COR Odyssey. Imaging parameters such as wavelength (nm) channel, intensity, and focus varied based on NIRF dye incorporated in the nanoemulsion.

### Cell viability

Raw 264.7 cells (P7-P10) were seeded at 5000 cells per well in a 96-well plate and incubated overnight at 37 °C and 5% CO_2._ Macrophages were activated using lipopolysaccharide (LPS, 500 ng/ml) for 18 h, followed by NE treatment for 24 h. According to the manufacturer's instructions, the CellTiter Glo 2.0 A Luminescent Cell Viability Assay Kit (Promega) was used to assess cell viability by quantifying ATP in metabolically active and viable cells. Using untreated macrophages as a control, the percentage of viable macrophages was calculated using Eq. (3).

3 $$\%Cytotoxicity=\frac{\left[A\right]control-\left[A\right]test}{\left[A\right]control} \, \times \,100$$

where [A]test is the absorbance of the test sample and [A]control is the absorbance of the control sample.

A membrane-based assay was performed on NE-treated cells using the CellTox Green Cytotoxicity Assay (Promega). Manufacturer instructions were followed for plate development. CellTox Green determines cell viability by quantifying the fluorescence signal created by nonviable cells with compromised membranes.

### High-performance liquid chromatography (HPLC)

CXB loading in nanoemulsions was measured using a validated reversed-phase HPLC method on a Dionex Ultimate 3000^87^. The method was based on isocratic elution of CXB using methanol–water (75:25) as the mobile phase on a C18 column (Hypersil Gold C18 150 mm × 4.6 mm, 5 µm pore size) with UV detection at 255 nm. The retention time for CXB was 3.8 min when the flow rate was maintained at 1 mL/min at 30 °C column temperature. For CXB content analysis, nanoemulsions were first diluted in pure methanol for CXB release. If the nanoemulsion contained PCE, NE dissolved methanol solution was then centrifuged for a few minutes (~ 1 min), allowing the undissolved perfluorocarbon to settle at the bottom. Methanol supernatants were then diluted in water to match the mobile phase ratio. All analyses were performed in triplicate. The HPLC standard curve for celecoxib with the limit of detection (LOD) and limit of quantification (LOQ) and representative HPLC chromatograph showing the CXB peak are shown in Supplementary Fig. 5B, C.

### In vitro celecoxib release study

The quantitative in vitro release test was performed at 37 ± 0.5 °C using the dialysis bag technique (molecular cutoff 3000 Dalton “Da”). A total of 1 mL of CXB dissolved in methanol (2 mg/mL, standard solution) and an equivalent drug containing CXB FA NE formulation was placed in the dialysis bag. The receptor compartment consisted of a 15 mL mixture of phosphate-buffered saline (PBS; pH 7.4) and methanol (4:1). To maintain sink conditions, the dialysis bag was switched into fresh medium in a 50 mL centrifuge tube at regular intervals (2, 5, 8, 24, 48, 72, 168, 366, and 528 h). The quantitative analysis of CXB in a 0.5 mL aliquot was performed using the HPLC method, as described in the “HPLC analysis” section. The cumulative percentage of drug release verses time was plotted to evaluate the drug release pattern from a CXB solution and the CXB FA NE.

### Enzyme-linked immunosorbent assay (ELISA)

Raw 264.7 cells (P7–P10) were seeded into 6-well plates at 0.3 million cells per well in FCCM medium. To maximize the pharmacological response, early cell passage number was used. Plates were incubated at 37 °C and 5% CO_2_ for 48 h. Cells were then treated for 24 h with CXB NE, DF NE, CXB FA NE, DF FA NE, free drug CXB, and free drug vehicle dimethyl sulfoxide (DMSO). Celecoxib dosages ranged from 40 μM, 10 μM and 2 μM intervals down half-log. DF NE and DMSO were volume-matched to CXB-loaded nanoemulsion and free drug CXB, respectively. After 24 h of incubation at 37 °C and 5% CO_2_ with treatments, all supernatant was removed from the wells. Raw 264.7 cells were activated using LPS (500 ng/ml) for 18 h in 37 °C and 5% CO_2_ incubation. The supernatant was then collected and spun at 4 °C and 1100 rpm for 5 min. Spun supernatants were stored at −80 °C until ELISA analysis. TNF-$\mathrm{\alpha }$ (Bio-Techne Corporation), PgE_2_ (Cayman Chemical Company), and IL-6 ELISA (Invitrogen) plate developments were all performed following the manufacturer’s protocol. Fluorescence measurements were obtained using a Synergy HTX plate reader (BioTek).

### ^19^F NMR measurement of PCE nanoemulsion cellular uptake

RAW 264.7 macrophages were plated at 0.5 × 10^6^ seeding density in a six-well plates, and allowed to attach overnight. Next day, 40 $\upmu$L of NE in 1 mL of cell culture media (labeling media) was added to the cells and left for 6 h of incubation at 37 °C. Later the labeling media was removed, cells were thoroughly washed with 1X PBS, detached by trypsinization, washed, and counted through a hemocytometer for cell number. Further, the cells were pelleted (~ 20 $\upmu$L) and were lysed through adding 180 $\upmu$L of deionized water. Lysed cells were transferred to NMR sample tube (7″ length, 4.1 mm inner diameter) (Wilmad-LabGlass, Vineland, NJ, USA). To this 200 μL 0.4% w/v trifluoroacetic acid (TFA) as an internal standard and 50 μL deuterium oxide was added and spectra were recorded by acquiring 512 scans for each sample (n = 3) (Bruker, 400 MHz). The number of ^19^F per cell (Fc) was calculated using the previously published approach^88^, NMR spectra were analyzed and plotted in MestreNova (Mestrelab Research).

### Fluorescence microscopy

Raw 264.7 cells were seeded at 20,000 cells per well (P10–P12) in 0.75 mL of cell culture media on an 8-well chamber slide system Lab-TekII. After approximately 24 h of incubation at 37 °C and 5% CO_2_, the cells were treated with 20 μL/mL dose of NE for 6 h_._ The treatments were removed, and the chamber slides were washed with warm 1× PBS and fixed with 4% paraformaldehyde at ambient temperature for at least 20 min and washed with 0.5 mL 1× PBS twice. After removing chamber wall, 2–3 drops of mounting media with DAPI were applied. A cover slip was placed gently to avoid bubbles and the slide was left in a dark place for 2–3 h for the DAPI nuclei staining. Images were taken on a Nikon NiU microscope with a Plan Apo 100X oil NA 1.45 objective and a Nikon DS-Qi2 digital camera controlled by Nikon Elements software. Confocal microscopy was carried out on a Nikon A1r Confocal Imaging system using the 405 nm and 640 nm lasers to image DAPI and NE (DiR) correspondingly, along with DIC images that were all acquired simultaneously with a Plan Apo λ 60X oil NA 1.40 objective. The instrument was controlled by Nikon Elements software scanning through the z-axis and rendered with maximum projection in 3D.

### Flow cytometry

For NE uptake, Raw 264.7 macrophages (P7-P10) were plated in 12-well plates (0.2 million cells per well) and left for attachment overnight in RPMI media without folate. Cells were exposed to LPS (500 ng/ml) for 18 h to activate macrophages. The day after aspiration of media, activated macrophages were treated with NEs (20 μL/mL) for 15 min, 30 min, 1 h, and 3 h. Resting macrophages were cultured in DMEM supplemented with 10% FBS. For activated macrophage uptake studies, cells were treated with cytochalasin B (5 μg/mL) or cytochalasin B with free folic acid (1 mM). NEs (20 μL/mL) were added to the existing medium for 3 h. Cells were collected by trypsinization and fixed at room temperature with 2% PFA in DPBS for 20 min. All experiments were performed in triplicate, and samples were analyzed using Attune Nxt (Thermofisher Scientific) recording 40,000 events. The nanoemulsion was detected in the RL3 (DiR, 748 nm/780 nm) channel. Gating was applied based on forward scatter (FSC) and side scatter (SSC), as shown in Supplementary Fig. 3.

### Statistical analysis

The data obtained were expressed in terms of mean $\pm$ standard deviation (SD) values. The data were analyzed by unpaired two-tailed Student’s t-test. The statistical significance level was set at p < 0.05, and all the data were analyzed using GraphPad Prism v9.3.1. For statistical analyses for the data presented refer to Supplementary Tables S1, S2, S3 and S4.

## Conclusion

We designed and developed an improved theranostic PFC NE formulation that can specifically target FR receptors expressed on activated macrophages. To the best of our knowledge, this is the first theranostic folate-conjugated PFC NE with established pharmacological efficacy in vitro. Folate-conjugated PFC NE (CXB FA NE) showed excellent colloidal and fluorescence stability. Furthermore, we evaluated this formulation in LPS-activated macrophages for an anti-inflammatory effect. Loading CXB in the NE platform showed an improved safety profile compared to the CXB solution. The addition of the targeting ligand FA increases uptake in activated macrophages compared to resting macrophages. The findings were able to show higher time-dependent uptake of CXB FA NE compared to CXB NE via folate receptors. Based on the in vitro findings, CXB FA NE showed anti-inflammatory action by suppressing PgE2, TNF-$\alpha$, and IL-6 release from LPS-activated macrophages in a dose-dependent fashion. Therefore, this formulation holds the potential to be tested further for in vivo studies*.* We also showed the feasibility of manufacturing theranostic NEs carrying three payloads (two dyes and a drug) on a large scale (200 mL). Based on the presented in vitro study, we conclude that folate-conjugated PFC NEs are promising anti-inflammatory theranostic nanosystems that are applicable to multiple inflammatory states and can be used in future preclinical studies. We also propose that these systems can serve as models for the development of future clinically viable theranostic nanomedicines.

### Supplementary Information

Supplementary Figures.

Supplementary Table S1.

Supplementary Table S2.

Supplementary Table S3.

Supplementary Table S4.

## Data Availability

Data will be made available by the corresponding author as per reasonable request.
