# Supplementary material for: Folate-conjugated near-infrared fluorescent perfluorocarbon nanoemulsions as theranostics for activated macrophage COX-2 inhibition
Source: Sci Rep. 2023 Sep 14;13:15229. doi: 10.1038/s41598-023-41959-9 (PMC10502124; doi:10.1038/s41598-023-41959-9)
Supplement: Supplementary file 1 — Supplementary Figures. [file 41598_2023_41959_MOESM1_ESM.pdf]

SUPPLEMENTAL INFORMATION

Folate-conjugated near-infrared fluorescent perfluorocarbon nanoemulsions as theranostics for activated macrophage COX-2 inhibition

*Riddhi Vichare[1], Caitlin Crelli[1], Lu Liu[1], Rebecca McCallin[1], Abree Cowan[1], Stefan Stratimirovic[1], Michele Herneisey[1], John A. Pollock[2] and Jelena M. Janjic[1]**

[1]Graduate School of Pharmaceutical Sciences, School of Pharmacy, Duquesne University, Pittsburgh, PA 15282, USA.

[2] Department of Biological Sciences, School of Science & Engineering, Duquesne University, Pittsburgh, PA 15282, USA

*Correspondence should be addressed to J.M.J [email: janjicj@duq.edu].

Supplemental Figures

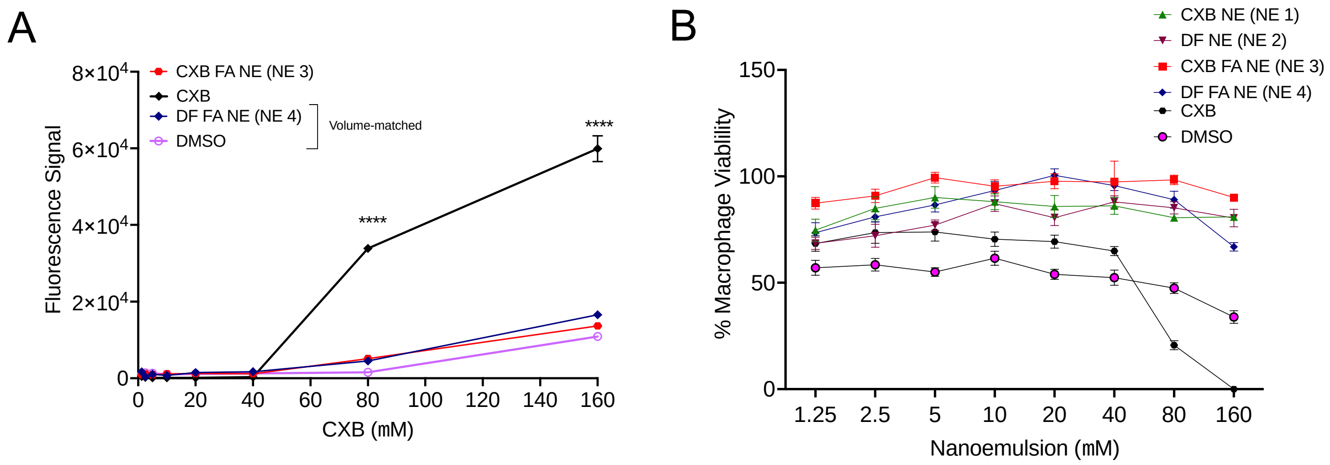

Supplemental Fig 1: A) Macrophages were exposed to CXB loaded NE 3 and NE 4, free drug solution: CXB in DMSO (10mM), and free drug vehicle: DMSO for 24h. Assay performed via cell membrane based CellTox Green. B) LPS-activated macrophages were exposed to NE 1, NE 2, NE 3, NE 4, CXB in DMSO, and free drug vehicle: DMSO. Assay performed via cell membrane based CellTox Green. The data points represent mean ± SD (n= 6).

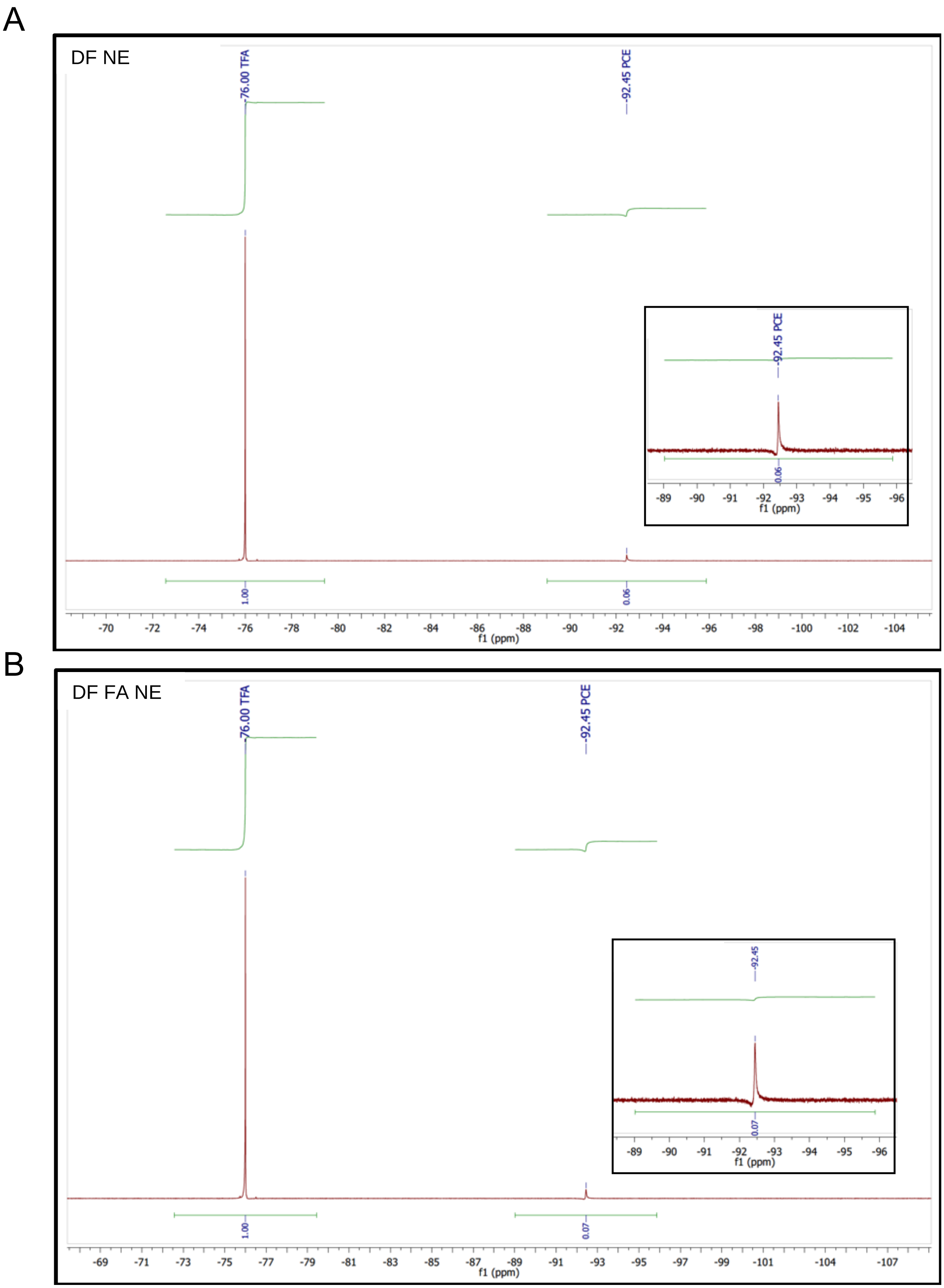

Supplementary Fig 2. A) Representative ^{19}F NMR spectrum of labeled RAW 264.7 macrophages with DF NE. B) Shows a ^{19}F NMR spectrum of labeled RAW 264.7 macrophages with DF FA NE. In both the spectra PCE (−92.45 ppm) and TFA (−76.00 ppm) reference.

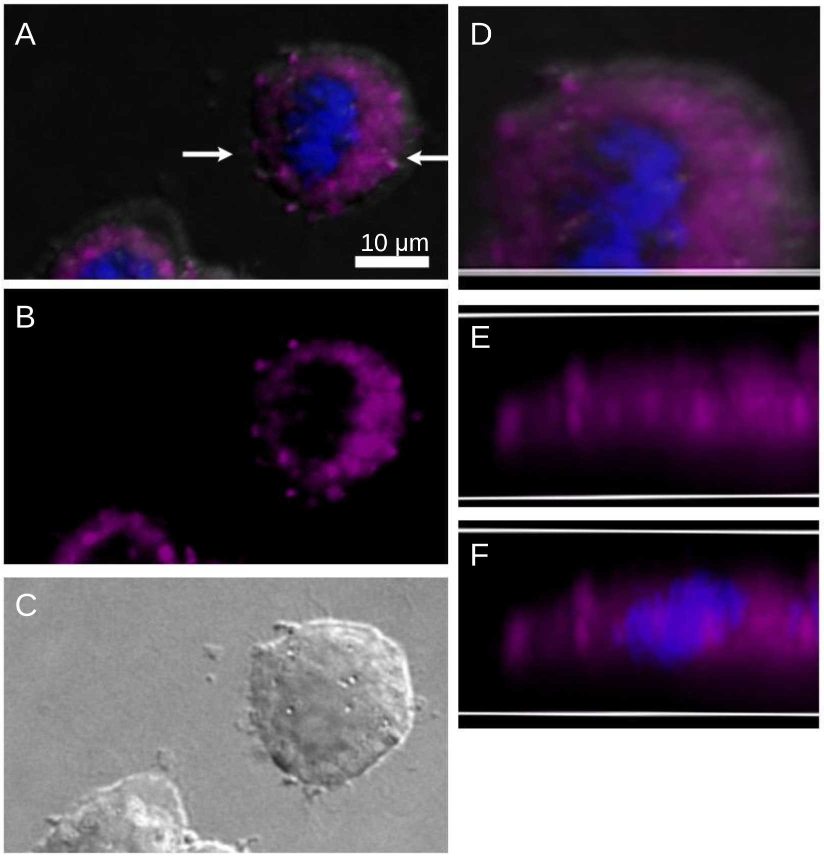

Supplementary Fig 3. Confocal microscopy reveals in 3D, the contents of the cell's cytoplasm containing particles/droplets of DiR nanoemulsion. A) Overlay of a 3D maximum projection of a z-series with DIC, DAPI, and DiR visualized. Arrow indicates the line for the 3D cross-section in (E) and (F). Bar = 10 µm. B) NIRF_DiR. C) DIC. D) 3D rendered volume projection, line at bottom is the level of cross-section. E) DiR rendered projected view through the cell. F) DiR / DAPI rendered projected view through the cell along the same line.

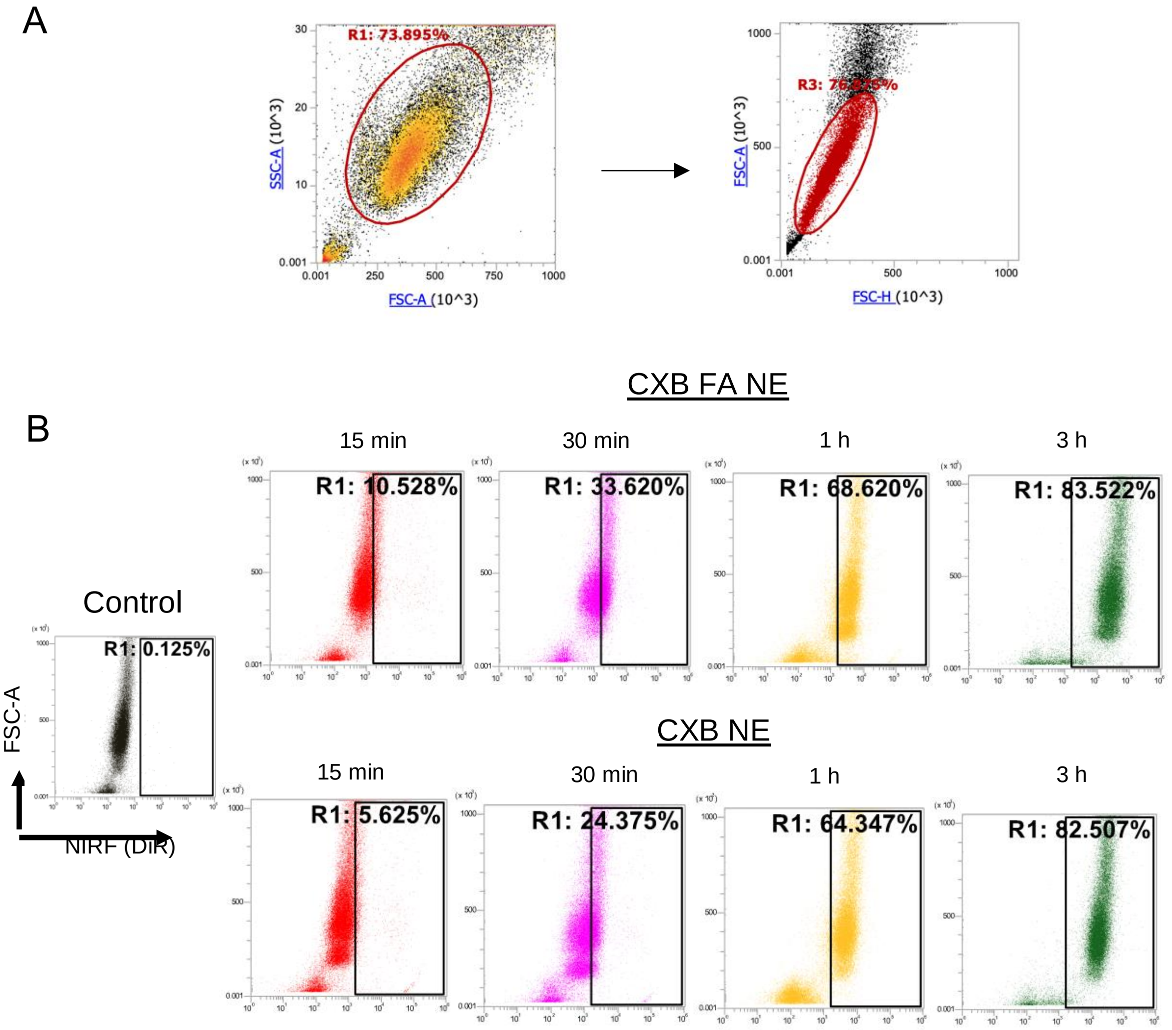

Supplemental Fig 4. A) All gating were first set on target cell population (FSC-A vs. SSC-A) followed by gating singlet events (FSC-A vs FSC-H). B) Dot-plots showing time-dependent (15 min, 30 min, 1h, 3h) cellular uptake of CXB NE verses CXB FA NE. (R1:DiR+).

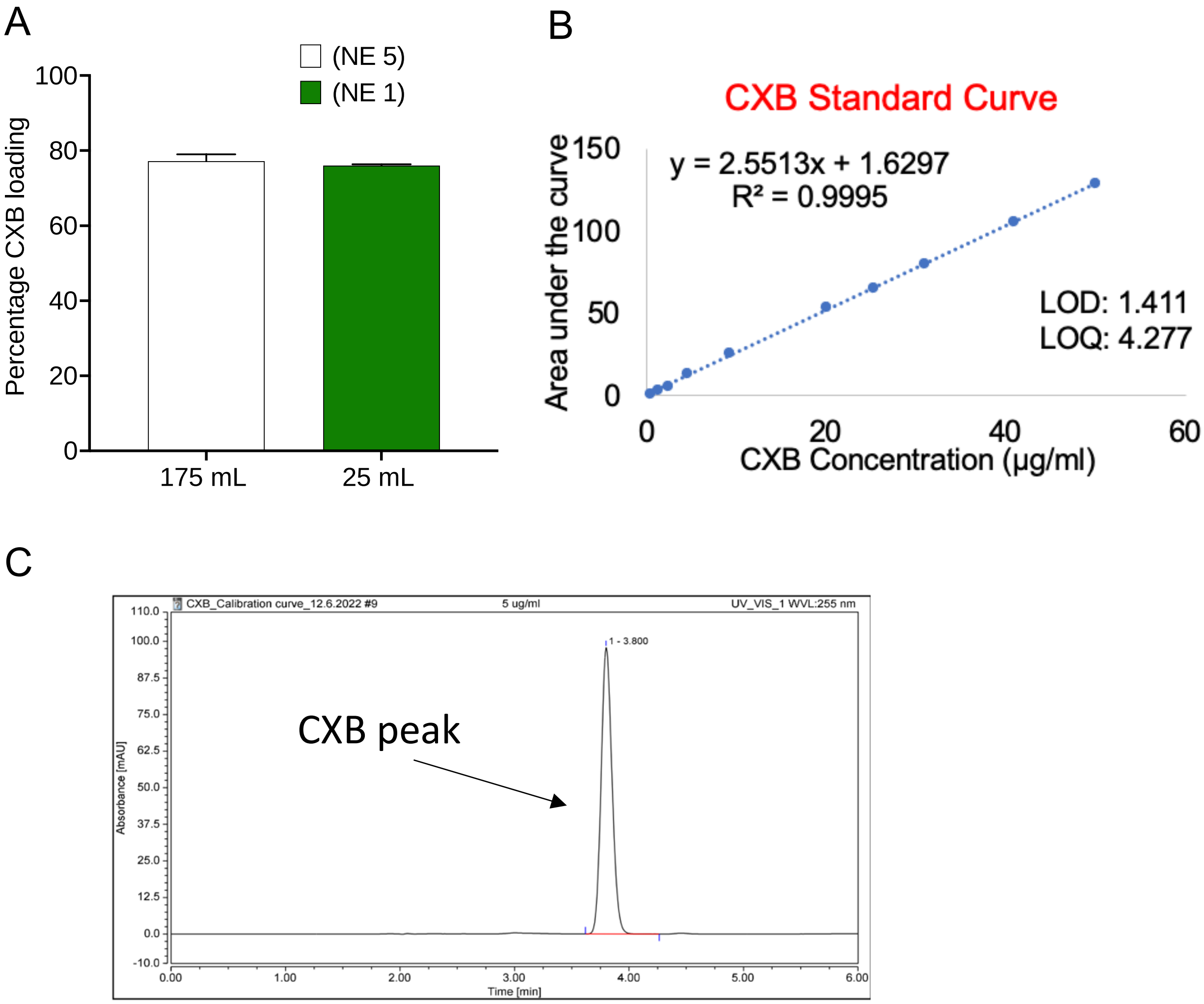

Supplemental Fig 5: A) Comparison of percent CXB loading in NEs between large-scale (NE 5) and small-scale (NE 1). B）HPLC method validation standard curve for celecoxib with limit of detection (LOD) and limit of quantification (LOQ). C) Representative of HPLC chromatograph indicating celecoxib drug peak using methanol-water (75:25) as the mobile phase on a C18 column with UV detection at 255 nm. The retention time for CXB was 3.8 min.

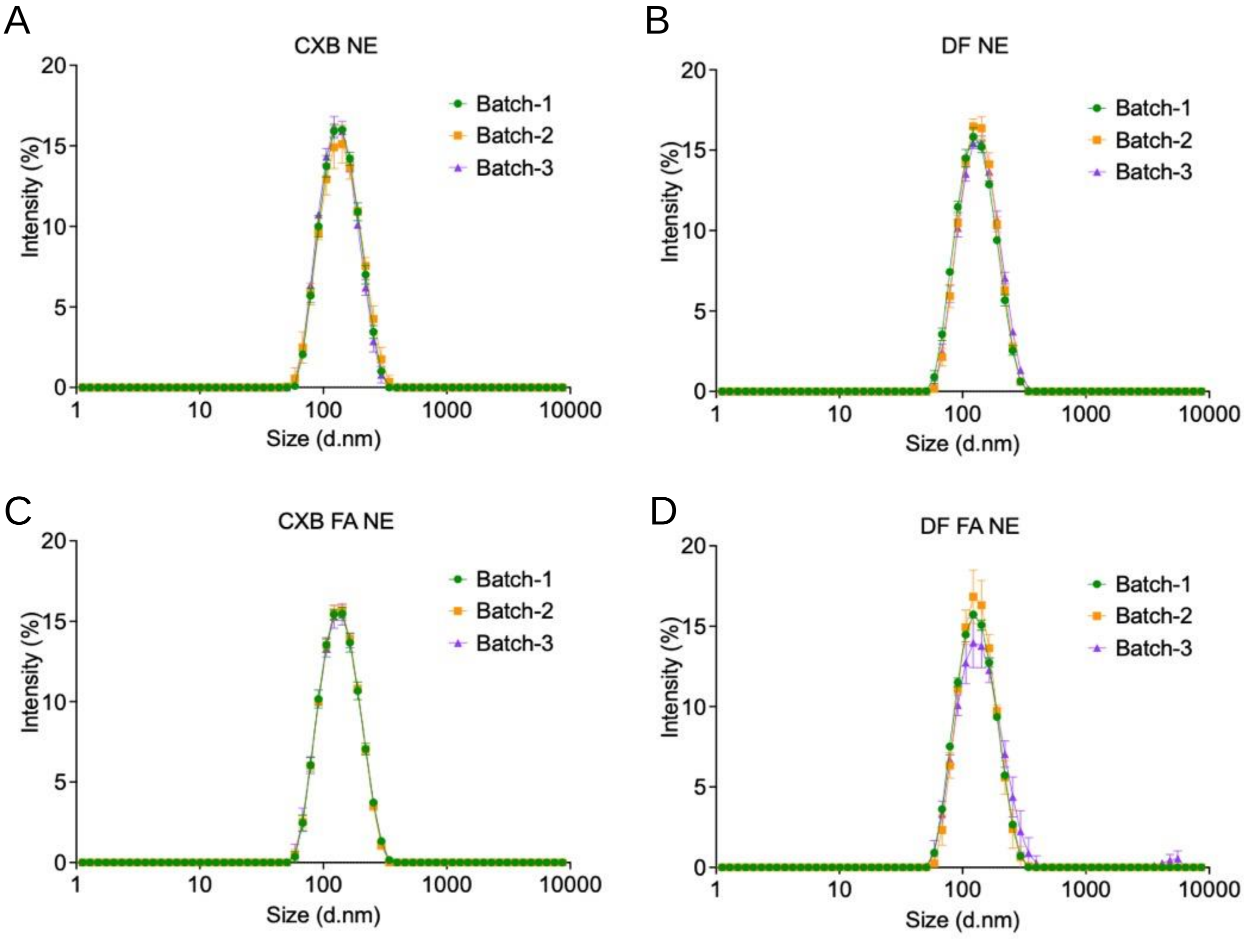

Supplemental Fig 6: A-D) Overlays of averaged size distributions from three reproducible batches of CXB NE, DF NE, CXB FA NE, and DF FA NE produced on M110S (25 mL).
