# Supplementary material for: Folate-conjugated near-infrared fluorescent perfluorocarbon nanoemulsions as theranostics for activated macrophage COX-2 inhibition
Source: Sci Rep. 2023 Sep 14;13:15229. doi: 10.1038/s41598-023-41959-9 (PMC10502124; doi:10.1038/s41598-023-41959-9)
Supplement: Supplementary file 2 — Supplementary Table S1. [file 41598_2023_41959_MOESM2_ESM.docx]

**Supplementary table S1:** Statistical comparison between CXB drug loading in NEs with and without folate (**Figure 1G**). Statistics generated through GraphPad Prism v9.3.1 software.

| Unpaired t test result | **Figure 1G** |
| --- | --- |
| P value | 0.6052 |
| P value summary | ns |
| One-or two-tailed P value | Two-tailed |
| t, df | t=0.5603, df=4 |
