# Supplementary material for: Folate-conjugated near-infrared fluorescent perfluorocarbon nanoemulsions as theranostics for activated macrophage COX-2 inhibition
Source: Sci Rep. 2023 Sep 14;13:15229. doi: 10.1038/s41598-023-41959-9 (PMC10502124; doi:10.1038/s41598-023-41959-9)
Supplement: Supplementary file 3 — Supplementary Table S2. [file 41598_2023_41959_MOESM3_ESM.docx]

**Supplementary table S2:** Statistical comparison on RAW 264.7 macrophage viability between CXB FA NE verses CXB solution (CXB in DMSO) (**Figure 4A**). Test: Multiple unpaired T tests. Statistics generated through GraphPad Prism v9.3.1 software.

| **Multiple unpaired t tests** | **Discovery** | **P value** | **Mean of CXB FA NE** | **Mean of CXB NE** | **Difference** | **SE of difference** | **t ratio** | **df** | **q value** |
| --- | --- | --- | --- | --- | --- | --- | --- | --- | --- |
| **1.25** | Yes | 0.010347 | 113.8 | 102.6 | 11.20 | 3.556 | 3.149 | 10.00 | 0.005225 |
| **2.5** | No | 0.348949 | 118.2 | 114.1 | 4.100 | 4.172 | 0.9827 | 10.00 | 0.132165 |
| **5** | No | 0.023157 | 115.5 | 106.6 | 8.900 | 3.323 | 2.678 | 10.00 | 0.010024 |
| **10** | Yes | 0.000074 | 121.7 | 108.3 | 13.40 | 2.079 | 6.445 | 10.00 | 0.000056 |
| **20** | Yes | 0.000179 | 117.3 | 102.9 | 14.40 | 2.494 | 5.773 | 10.00 | 0.000109 |
| **40** | Yes | 0.000013 | 119.6 | 94.00 | 25.60 | 3.233 | 7.918 | 10.00 | 0.000013 |
| **80** | Yes | <0.000001 | 117.1 | 33.60 | 83.50 | 1.666 | 50.11 | 10.00 | <0.000001 |
| **160** | Yes | <0.000001 | 107.7 | 0.1000 | 107.6 | 1.878 | 57.30 | 10.00 | <0.000001 |
