# Supplementary material for: Folate-conjugated near-infrared fluorescent perfluorocarbon nanoemulsions as theranostics for activated macrophage COX-2 inhibition
Source: Sci Rep. 2023 Sep 14;13:15229. doi: 10.1038/s41598-023-41959-9 (PMC10502124; doi:10.1038/s41598-023-41959-9)
Supplement: Supplementary file 4 — Supplementary Table S3. [file 41598_2023_41959_MOESM4_ESM.docx]

**Supplementary table S3:** Statistics on quantitative analysis performed by flowcytometry on (LPS)-activated RAW 264.7 macrophages. Macrophage uptake compared between DiR-labelled CXB NE and CXB FA NE (**Figure 5A**). Test: Unpaired T tests. Statistics generated through GraphPad Prism v9.3.1 software.

| **Unpaired t test** | **Figure 5A** |
| --- | --- |
| CXB FA NE vs CXB NE | **30 min** |
| P value | 0.0031 |
| P value summary | ** |
| Significantly different (P<0.05)? | Yes |
| One- or two tailed P value? | Two-tailed |
| t, df | t=6.354, df=4 |
| **Unpaired t test** |  |
| CXB FA NE vs CXB NE | **60 min** |
| P value | 0.0216 |
| P value summary | * |
| Significantly different (P<0.05)? | Yes |
| One- or two tailed P value? | Two-tailed |
| t, df | t=3.661, df=4 |
