# Supplementary material for: Folate-conjugated near-infrared fluorescent perfluorocarbon nanoemulsions as theranostics for activated macrophage COX-2 inhibition
Source: Sci Rep. 2023 Sep 14;13:15229. doi: 10.1038/s41598-023-41959-9 (PMC10502124; doi:10.1038/s41598-023-41959-9)
Supplement: Supplementary file 5 — Supplementary Table S4. [file 41598_2023_41959_MOESM5_ESM.docx]

**Supplementary table S4:** Statistical comparison of the pharmacological responses of NEs with or without folate and CXB solution on activated macrophages (**Figure 7**). Test: Unpaired T test. Statistics generated through GraphPad Prism v9.3.1 software.

| **TNF-**$\boldsymbol{\alpha}$ | p value |
| --- | --- |
| CXB FA NE vs CXB solution(40$\mu$M) | 0.0096 |
| CXB FA NE vs +LPS | 0.0176 |
| CXB FA NE vs CXB NE (40$\mu$M) | 0.0016 |

| **IL-6** | p value |
| --- | --- |
| CXB FA NE vs CXB solution (40$\mu$M) | 0.011 |
| CXB FA NE vs +LPS | 0.0010 |
| CXB FA NE vs CXB NE (40$\mu$M) | 0.0016 |

| **COX-2** | p value |
| --- | --- |
| CXB FA NE vs CXB solution (40$\mu$M) | 0.0096 |
| CXB FA NE vs +LPS | 0.0004 |
| CXB FA NE vs CXB NE (40$\mu$M) | Not significant |
